# Working memory span tasks: Group administration and omitting accuracy criterion do not change metric characteristics

**DOI:** 10.1371/journal.pone.0205169

**Published:** 2018-10-11

**Authors:** Ratko Đokić, Maida Koso-Drljević, Nermin Đapo

**Affiliations:** Department of Psychology, Faculty of Philosophy, University of Sarajevo, Sarajevo, Bosnia and Herzegovina; Columbia University, UNITED STATES

## Abstract

The study examined the psychometric characteristics of three automated working memory span tasks: operational, reading, and symmetry span task, administered in groups of participants. For each task, the metric characteristics of six scoring procedures were evaluated: partial, absolute, partial non-weighted, absolute non-weighted, partial weighted, and absolute weighted scoring. Metric characteristics of all measures were compared across two parallel analyses: with and without application of a typical 85% accuracy criterion on the processing component of the tasks. The study demonstrates that the group administration of span tasks does not compromise their psychometric characteristics. All the tasks had an adequate internal consistency with Cronbach’s *α*s equal to or above .70; the exception being all types of the symmetry span task absolute scores with *α* values close to .60. Furthermore, all tasks have satisfactory convergent construct validity as well as criterion validity estimated in relation to measures of fluid intelligence. Omitting the 85% accuracy criterion on the processing component of the span tasks did not impair their psychometric properties. Thus, it is recommended that researchers discard this accuracy criterion as a criterion for filtering the results for further statistical analyses.

## Introduction

By developing further the concept of the central executive from Baddeley and Hitch’s working memory (WM) model [1], Engle and colleagues proposed a dual-component model of working memory capacity (for an overview, see: [2–6]). Within the dual-component model, working memory capacity (WMC) is defined by two controlled functions of the central executive that surface in the conditions of interference. The first function is executive attention, i.e. one’s capability to direct attention within the domain of primary memory in order to maintain relevant information in a highly activated state in the presence of interferences [3]. The second function is a strategic search of secondary memory, which reflects the capability to retrieve relevant information from longer-term storage, in the presence of competitive, irrelevant information [5,6]. This twofold nature of the WMC is reflected in the structure of WM span tasks (span tasks). In span tasks, in between presentations of the individual to-be-remembered items (e.g. individual letters), which comprise the memory component of the tasks, participants are engaged in processing portion of the tasks (e.g. solving simple mathematical equations). In that way, WMC–operationalized as the number of to-be-remembered items recalled in correct serial order–is measured in conditions of interference caused by the second, processing component of the task. At the conceptual level, individual differences in WMC have two main sources. The first is an individual’s ability to maintain the relevant information in the focus of attention. If this is not possible (for example, because of interferences triggered by the processing component of the span task) this information needs to be efficiently recalled from the secondary memory in the presence of concurrent information (see: [5–7]). Thus, the ability to retrieve information from secondary memory comprises the second source of variability in WMC.

To a large extent, it is the psychometric soundness of span tasks (see [7–9]), as well as their availability (see the web page of the Attention & Working Memory Lab, Georgia Institute of Technology, [10]), which explain why the dual-component model of WMC has been extensively empirically confirmed. Keeping in mind that span tasks are amongst most widely used measures in cognitive psychology today, the issue of advancing the effectiveness of their administration is constantly present. Thus, automation of tasks has enabled participants to perform them independent of the experimenter, and made the recording and processing of the data significantly simpler. At the same time, the tasks have been methodologically improved by setting individualized time limits in the processing component in order to restrict the participants from rehearsing to-be-remembered items during the processing tasks [9]. Lately, the focus is on shortening span tasks in order to shorten the time necessary for their administration [11,12].

Accordingly, our attempt at improving the efficiency of span task utilization concerns the assessment of their psychometric characteristics in group administration. Specifically, we were interested in examining whether the group administration of three span tasks–the operational span task (OSPAN), the reading span task (RSPAN), and the symmetry span task (SSPAN)–undermines their psychometric properties compared to the normative data established during individual administrations of the tasks [8,9]. Psychometric indicators of primary interest were the internal consistency of the WMC measures, their convergent construct validity, as well as their criterion validity related to measures of fluid intelligence (*g*F), both on the task and latent level. *g*F was selected as a criterion measure because, from the perspective of cognitive psychology, WMC is especially interesting both as a consistent predictor and as one of the theoretical presuppositions of reasoning (e.g. [13–20]).

Another, equally important goal of the study was to examine if there is possibility of reducing the portion of participants whose WMC results are excluded from further analyses. Namely, in a typical research procedure only the results of participants who achieve the 85% accuracy criterion on the processing component of the span task are taken into analysis. This criterion is to ensure that participants were truly engaged in solving the processing tasks (which, according to the dual-component model of WMC, creates interference in maintaining and recalling the to-be-remembered items). Hence, Unsworth et al. [9] reported that 15% of participants in their study were excluded from the analysis for failing to achieve the 85% accuracy criterion on the OSPAN processing component. However, Unsworth, Redick, et al. [21] concluded that the 85% accuracy criterion is unnecessary; this conclusion was based on the observation of the correlation between the results on the processing and memory component of span tasks, that is, on the pattern by which participants that are faster and more successful at solving the processing tasks, also reproduce more to-be-remembered items. In order to investigate more thoroughly the necessity of the accuracy criterion, our approach in this study was to gradually decrease it from an initial 85% through 80% and 75% to 50% accuracy, and finally to its complete exclusion. If proven justified, reducing or even completely omitting of the 85% accuracy criterion on the processing component of the tasks would significantly improve the effectiveness of future research, as the total number of examinees would be lower, and time would be saved.

In the study, we considered the psychometric characteristics of six different WMC scores, extracted by each of the three span tasks. Namely, the span tasks are comprised of sets in which processing tasks and to-be-remembered items are sequentially interchanged. The sets consist of three to seven sequences in OSPAN and RSPAN, and two to five sequences in SSPAN. In all the tasks, sets of varying lengths are presented three times in random order. Such a design enables the calculation of six different WMC scores. These scores are: partial, absolute, partial non-weighted, absolute non-weighted, partial weighted, and absolute weighted score. Differences between the scoring procedures are determined according to two dimensions. The first one refers to whether or not the scoring procedure takes into account all the sets, or only those that were completely correctly reproduced. Partial scoring counts all to-be-remembered items recalled in the correct serial position, regardless of whether they come from completely or only partially correctly recalled sets. In contrast, absolute scoring counts to-be-remembered items only from completely correctly reproduced sets. The second dimension concerns non-weighted as opposed to weighted scoring. With non-weighted scoring procedures, all to-be-remembered items are treated equally, regardless of the length of the set that they belong to. That is achieved by firstly calculating the proportion of correctly recalled items within a set, and then by expressing the final result as an average proportion for all sets combined. In weighted scoring procedures, longer sets contribute more to the final result. That is achieved by expressing the weighted score as the average of all the correctly recalled items (regardless of the length of the set that the items belong to). For a more detailed discussion on span tasks scoring procedures, please see [7].

Conclusions from previous studies prefer partial to absolute scoring procedures [8,22]. In partial procedures, all available information is taken into consideration, i.e. partially recalled sets are not disregarded. In this way, partial procedures are more sensitive compared to absolute procedures. Namely, partial scores express the difference between participants in units of correctly reproduced individual items, rather than in units expressing the length of correctly reproduced sets. In other words, absolute scoring does not discriminate participants who reproduce almost all items in a given set (e.g. six out of seven) from those who recalled some or no items in the same set (e.g. one out of seven): in both cases, the absolute score is zero. In that way, partial scoring procedures, compared to absolute scoring procedures, provide more continuous measures and, due to better detection of individual differences, provide better characteristics of distribution and reliability [22]. We were interested in whether these conclusions would hold also when the span tasks were administered in a group setting.

In addressing the above questions, this study represents a large scale investigation of whether group administration, scoring procedures, and accuracy criterion influence the psychometric properties of three common span tasks. The results were, in outline, that group administration of the tasks gave results similar to those typically found with individual administration. The results also suggest that partial scoring procedures are to be preferred to absolute scoring procedures, and further suggest that adhering to a strict 85% accuracy criterion is not necessary, as omitting the criterion leads to comparable results. As the analyses with different accuracy criteria– 85%, 80%, 75%, 50%/ no accuracy criterion–yielded similar results, this paper presents only the results obtained with the 85% accuracy criterion and with no accuracy criterion applied, while analyses with other listed criteria are presented in S1–S8, S10 and S11 Tables.

## Method

### Ethics statement

The study was reviewed and approved by the Department of Psychology, Faculty of Philosophy, University of Sarajevo. [In Bosnia and Herzegovina, there are no ethics committees responsible for approving (or waiving the necessity of ethical approval of) psychological research involving human subjects, neither at national nor at institutional level.]

Participation of the participants in the study was part of their regular academic activities included in the academic syllabus presented to the students at the beginning of the academic semester. All participants were informed about the nature and the aim of the study in advance. Participants were also informed that participation in the study was voluntary and that they were not obliged to participate; it was also explained that they could leave the study at any point. Participants received course credits for their participation; they were informed that they could get course credits for another academic activity in case they did not want to participate in the study. Participants participated in the study under a unique ID code. To document verbal consent for the participation in the study, the participants recorded their ID code at the beginning of the testing. Written consent was not deemed necessary as participation in the study was part of the participants’ regular academic activity and because the data was anonymized. All interested participants were able to get feedback information on their results on the applied intelligence tests. These procedures are in compliance with the norms for research on human subjects in the Ethical Codex of the University of Sarajevo and Ethical Codex of the Psychological Society of the Federation of Bosnia and Herzegovina.

### Participants

In the study, for a total of 504 participants (82% female) results on at least one span task were collected. Participants were between the ages of 18 and 41 (*M* = 19.67, *SD* = 1.77), and at the time of testing they all were first year students at the University of Sarajevo, Department of Psychology. All participants were native Bosnian speakers.

### Tasks

#### Automated working memory (WM) span tasks

The automated span tasks validated in this study are an adapted version of the original tasks programmed in the *E-Prime 2*.*0 Professional* software [23], and downloaded from [10]. A detailed description of the tasks can be found in [8,9,21]. All tasks were translated into Bosnian.

OSPAN. The participants were instructed to solve a simple mathematical equation, and verify the solution offered, as fast as possible. After that, the participants were presented a letter to be memorised. The letter was one from the following: F, G, H, K, L, N, P, R, S, T, V, and Z. Immediately following the presentation of the letter, the program presented the next equation. A set could contain from three to seven sequences in which letters and equations alternate. At the end of each set, the participants were instructed to recall the letters in the correct serial order by clicking the appropriate letters on the computer screen. Sets of different sizes were presented three times in random order, for a total of 75 equations and letters.

After each set, the participants were given a feedback on the number of recalled letters, the number of incorrectly solved equations, as well as on the percentage of successfully solved equations during the entire experiment. Instructions emphasized that during the entire experiment, participants needed to maintain an accuracy in solving the processing tasks of above 85%. Feedback on the success in the memory and processing component of the task, as well as emphasizing the importance of the 85% accuracy criteria was common to all three span tasks.

Prior to the beginning of the experimental session, participants completed three training blocks that consisted of (a) the memory component of the task alone, (b) the processing component of the task alone, and (c) processing tasks in between which the to-be-remembered items were presented, imitating the experimental session. Time available for solving the processing tasks in the experimental session was individualised as the average time (plus 2.5 *SD*) that was needed for solving the processing tasks in the training block (b) for that individual. If the participants exceeded this time limit for solving the processing task, the program automatically proceeded with the presentation of the next letter and counted that trial as an error. The same procedures of training and individualisation of time available for solving the processing portions of tasks were applied in other two span tasks as well.

RSPAN. RSPAN had the same structure as the OSPAN with the only difference that the participants assessed whether sentences which were presented made sense or not, instead of solving mathematical equations. The participants were instructed to, as fast as possible, read the presented sentence and indicate whether it made sense or not. After that, the participants were presented with a letter to be memorised (to-be-remembered letters were the same as in OSPAN). Immediately following the presentation of the letter, the program presented the next sentence. Each set contained from three to seven sequences in which letters interchanged with sentences. At the end of each set, the participants were instructed to recall the letters in the correct serial order by clicking the appropriate letters on the computer screen. Sets of different sizes were presented three times in random order, for a total of 75 sentences and letters.

The sentences used in RSPAN were created in accordance with the original sentences used in the US-version of the task. All sentences were loosely translated from English into Bosnian; the original sentences that could not be adjusted to the specific characteristics of the Bosnian language were replaced by completely new sentences. Half of the presented sentences made sense. Nonsensical sentences were made by replacing a word in otherwise meaningful sentences (e.g. “The prosecutor’s dish was lost because it was not based on fact.”). After the translation, a professional proof-reader verified all the sentences in order to ensure their grammatical accuracy and semantic (in)adequacy. All sentences in the experimental section of the task contained between 10 and 15 words.

SSPAN. The participants were instructed to indicate, as fast as possible, whether a presented matrix was symmetrical, while at the same time trying to memorise the locations of red squares in a grid. In the symmetry-judgment processing task, the participants were presented with the 8 x 8 matrix, containing some black cells. The participants estimated whether the matrix was symmetrical about its vertical axis. Matrices were symmetrical in half of the presentations. Immediately after indicating whether a matrix was symmetrical or not, the participants were presented with a 4 x 4 grid containing one red square. After the presentation of the red square, the program presented the next matrix. A set could contain between two and five sequences, in which squares and matrices alternated. At the end of each set, the participants were instructed to recall the locations of the red squares in the correct serial order by clicking on the cells of an empty grid on the screen. Sets of different sizes were presented three times in random order, for a total of 42 matrices and squares.

At the end of each span task, five scores were presented: (a) partial score (PSC), representing the sum of all items recalled in the correct serial order, regardless of whether the entire set was recalled correctly; (b) absolute score (ASC), summing only the elements from completely recalled sets; (c) accuracy errors, which is the number of incorrectly solved processing tasks; (d) speed errors, or the number of processing tasks unsolved before the individualised time limit expired; (e) processing errors, or the total number of errors on the processing component, i.e. the sum of speed and accuracy errors.

These scores than became the basis for calculating four additional WMC scores [7]. Non-weighted scores–a partial non-weighted score (PNS) and an absolute non-weighted score (ANS)–are the average proportion of correctly recalled to-be-remembered items across all sets. Calculating the proportions of correctly recalled to-be-remembered items within individual sets ensures that each set, regardless of its length, contributes equally to the final score. The difference between the two scoring procedures, once again, is whether all the correctly reproduced to-be-remembered items (i.e. PNS) or only items from completely recalled sets (i.e. ANS) are taken into account.

On the other hand, the weighted scoring procedures–partial weighted score (PWS) and absolute weighted score (AWS)–are simple linear transformations of PSC and ASC, respectively. Namely, these procedures sum up the correctly recalled to-be-remembered items, whether from all (i.e. PWS) or only completely recalled sets (i.e. AWS), and express the sum as a proportion of the total number of items presented. These procedures, just like PSC and ASC, put more weight on the items belonging to longer sets by leaving out the information on the length of individual sets from the final score equation.

[In order to make an easier comparison of terminology, Conway et al. [7] use the following terms and abbreviations for WMC scores: partial-credit unit scoring (PCU) for PNS, all-or-nothing unit scoring (ANU) for ANS, partial-credit load scoring (PCL) for PWS, and all-or-nothing load scoring (ANL) for AWS.]

#### Fluid intelligence (*g*F) tasks

Raven Advanced Progressive Matrices (Raven). The Raven is paper-and-pencil measure of abstract reasoning [24]. The test consists of 36 problems presented in order, from the simplest to the most complex. Each problem consists of 3 x 3 matrix of geometric patterns with the bottom right pattern missing. The task for the participants is to choose from eight alternatives the pattern that correctly completes the matrix. The final score was the total number of correctly solved problems. The test lasted 40 minutes. Prior to the beginning of the test, the participants completed a five-minute long exercise which consisted of 12 practice problems.

Verbal General Ability Test (VGAT). The test consists of 36 word-analogy problems. In each problem, participants are presented an incomplete analogy. The task for the participants is to choose from six alternatives the word that correctly completes the analogy [25]. The test lasted 15 minutes. Prior to the beginning of the test, the participants completed a four-minute long exercise which consisted of four practice problems.

Numerical General Ability Test (NGAT). The test consists of 36 problems divided into two groups: analogies and series. In all the problems, numbers are placed in a matrix missing one or two numbers. The participants’ task is to resolve the rule by which numbers in the matrix are interrelated, and then to choose among six alternatives, the number (or pair of numbers) that correctly completes the matrix. In order to exclude the influence of arithmetic ability to the result of the test, all problems include only four basic arithmetic operations involving integers [25]. The test lasted 20 minutes. Prior to the beginning of the test, the participants completed a five-minute long exercise which consisted of four practice problems.

### Procedure

Data were collected in three test sessions. Different test sessions were separated by at least one week and at most two months. In the first two sessions, intelligence tests were administered in groups from 45 to 80 participants. In the first session, which lasted about 50 minutes, participants completed the Raven. In the second session, which lasted about 45 minutes, participants completed VGAT and NGAT. All participants completed the intelligence tests in the aforementioned order (first Raven, than VGAT, and NGAT).

Within the third test session, automated span tasks were administered in groups from two to four participants (the majority of groups consisted of four participants). The participants were seated at computers positioned in four corners of a room; thus, each participant faced the other participants either sideways-on or back-to-back. The experimenter was present in the room together with the participants throughout the whole session. The task instructions were displayed on the computer screens in front of the participants. At the same time, the experimenter read the instructions out loud. At the beginning of the session, participants were instructed not to click the mouse to advance to the next screen until the experimenter had finished reading the current instruction. Also, participants were instructed not to start the experimental task even after having read instructions and completing the training segments, until the experimenter told them to. This procedure ensured the same tempo through the instructions and the training segments for all the participants, and also ensured that they all started the experimental task at the same time. The aim of this procedure was to reduce the distraction that could be introduced by the presence of other participants. In addition, the participants were asked to remain seated if they finished the tasks before the other participants, in order not to disturb them.

The order of the span tasks was counterbalanced across groups using a Latin-square design. The administration of the span tasks lasted about an hour per group (20 to 25 minutes for completion of OSPAN or RSPAN; 15 to 20 minutes for completion of the SSPAN). Participants did not have a break between span tasks.

## Results and discussion

Of the initial 504 participants, two forgot to bring their spectacles to testing; testing for two participants of OSPAN and SSPAN was terminated because of distractions, whereas data for four participants of SSPAN, three of OSPAN, and two of RSPAN were not saved due to the experimenter’s error. Due to computer malfunction, individualised times for the processing component at RSPAN were not set for two participants. One participant started performing SSPAN before the other participants. The results of these participants on the named span tasks were excluded from further analyses.

In Table 1, for each span task, the structure of the subsample of participants who accomplished the 85% accuracy criterion on the processing component of the span task is compared to the full sample in which no accuracy criterion was applied.

**Table 1:** Structure of the (sub)samples according to the working memory (WM) span task and the accuracy criterion on the processing component of the task.

Accuracy criterion	Subsample of participants above the accuracy criterion			% of participants below the 85% accuracy criterion
	n (% female)	Age		
		*M*	*SD*	
OSPAN: *N* = 497 (82.09)^a^				
85%	428 (81.54)	19.61	2.31	13.88
No accuracy criterion	497 (82.09)	19.66	1.77	-
RSPAN: *N* = 498 (82.13)^a^				
85%	484 (81.82)	19.60	2.18	2.81
No accuracy criterion	498 (82.13)	19.67	1.76	-
SSPAN: *N* = 495 (82.22)^a^				
85%	382 (80.63)	19.56	1.70	22.83
No accuracy criterion	495 (82.22)	19.66	1.76	-

*Note*. OSPAN = operation span; RSPAN = reading span; SSPAN = symmetry span.

^a^Total number of participants tested on the WM span task (% female).

The largest number of participants succeeded in fulfilling the 85% accuracy criterion on RSPAN, which is followed by OSPAN and then SSPAN. Thus, the percentage of examinees who failed to achieve the standard 85% accuracy criterion on the processing component of RSPAN was 3% (14 out of 498 participants). For OSPAN, that percentage was 14% (69 out of 497 participants), which corresponds to 15% of participants who failed under the same criteria in Unsworth et al. [9]. The percentage of participants who failed to meet the 85% accuracy criterion on SSPAN was a high–and arguably unsustainable– 23% (113 out of 495 participants). It seems that the symmetry-judgment task, as the processing component in SSPAN, was more demanding for our participants than solving the math equations in OSPAN, and especially more demanding than judging the sense of sentences in RSPAN.

### Descriptive statistics

Descriptive statistics for WMC scores and processing errors for each span task, according to the relevant accuracy criterion, are presented in S2 Table.

All WMC scores for all the span tasks, both in the 85% accuracy subsample and in full sample with no accuracy criterion applied, were approximately normally distributed (skewness <2 and kurtosis <4; [26]). Among all storage measures, the highest values of skewness (-1.07) and kurtosis (1.26) were observed for the OSPAN PNS distribution with no accuracy criterion applied.

For each span task, the means and standard deviations of particular WMC scores are remarkably similar, regardless of whether accuracy criteria were applied to the processing component of the task or not.

On the other hand, when omitting the 85% accuracy criterion, the distributions of processing errors (i.e. accuracy errors, speed errors, and total processing errors) transformed from normal into positively skewed and leptokurtic (S2 Table). This means that, by excluding the accuracy criterion, the sample extended to include a small portion of participants with an extreme number of errors; that made the distributions of processing errors skewed to the right and leptokurtic. In other words, our participants, as a rule, tried to keep the number of processing errors to a minimum while completing the span tasks. For a more thorough discussion of the distributions of errors on span tasks, please see [8].

Correlations between all WMC measures and the number of processing errors for all tasks and regardless of the accuracy criterion are negative and statistically significant at the *p* <.01 level (S3 Table). Thus, as a rule, the participants who achieved a better result (i.e., made fewer mistakes) on the processing components of span tasks also achieved a better result on the memory components (i.e., recalled more to-be-remembered items). The direction of correlations between results on the two components of span tasks reduces the possibility that the participants were using strategies to achieve a better result on the memory component to the expense of the processing component. Thus, this is an additional finding that challenges the common practice of using the 85% accuracy criterion as a criterion for filtering the WMC results for further analyses (see also [21]).

### Internal consistency

Cronbach’s *α* coefficients of the internal consistency of the WMC measures were calculated on the basis of three subscales of scores. The first subscale was created as a simple combination of scores from the first presentation of sets of all lengths; the two remaining subscales were created by combining the scores from the second and the third presentation of sets, respectively (for the details of the procedure, see [15,9]).

All OSPAN and RSPAN scores in both (sub)samples have *α* coefficients of .75 or higher (Table 2), which suggests adequate reliability for these measures. For OSPAN, the *α* coefficients range from .76 (for ASC/ AWS with 85% accuracy criterion) to .83 (for PNS with no accuracy criterion). For RSPAN, the *α* coefficients range from .75, for all absolute measures in both (sub)samples, to .80, for all partial measures–again–in both (sub)samples.

**Table 2:** Cronbach’s *α* for six working memory capacity (WMC) scores according to the working memory (WM) span task and accuracy criterion on the processing component of the task.

Measure	Accuracy criterion	OSPAN	RSPAN	SSPAN
		(n = 428/ 497)^a^	(n = 484/ 498)^a^	(n = 382/ 495)^a^
PSC/ PWS^b^	85%	.810	.801	.695
	No acc. crit.	.823	.804	.743
ASC/ AWS^b^	85%	.762	.754	.624
	No acc. crit.	.775	.753	.654
PNS	85%	.817	.802	.699
	No acc. crit.	.831	.805	.753
ANS	85%	.773	.748	.611
	No acc. crit.	.783	.747	.649

*Note*. OSPAN = operation span; RSPAN = reading span; SSPAN = symmetry span; PSC = partial score; ASC = absolute score; PNS = partial non-weighted score; ANS = absolute non-weighted score; PWS = partial weighted score; AWS = absolute weighted score; No acc. crit. = no accuracy criterion.

^a^Size of the subsample of participants above the 85% accuracy criterion/ size of the full sample with no accuracy criterion.

^b^As PWS is a simple linear transformation of PSC, their *α*s are identical. The same holds for AWS and ASC.

Reliabilities for SSPAN scores are generally lower than those for OSPAN and RSPAN, which is a trend also observed by Redick et al. [8]. Thus, the reliability coefficients for all SSPAN partial scores, regardless of the accuracy criterion, are in the range of the commonly used criterion of .70 (see [7,8]): the lowest is .70 and the highest is .75. For all types of SSPAN absolute scores, the *α* values are of the magnitude of .60: the range is from .61 to .65 (Table 2).

Coefficient *α*s of WMC scores observed at the 85% accuracy criterion in our study are somewhat lower than the normative values in Redick et al. [8]. At the descriptive level, coefficients for SSPAN deviate the most from normative values: by .12 for PSC and by .11 for ASC. Deviations in RSPAN are .08 for both PSC and ASC. In OSPAN, deviations from normative values are .05 for PSC and .04 for ASC.

All span tasks in all subsamples have somewhat higher reliability coefficients for partial than for absolute scores (for the same finding, see [7,8]). Differences in *α* values between two types of scores are somewhat higher for SSPAN than for OSPAN and RSPAN.

Omitting the accuracy criterion in the processing component did not weaken the reliability of span task scores (on the contrary, with the exclusion of the accuracy criterion, all *α* values slightly increased). Thus, according to the internal consistency criterion as well, it appears that the standard of at least 85% accuracy on the processing component is too strict a condition for ensuring adequate psychometric properties of span tasks.

### Correlations among different working memory capacity scores (WMC) of the same span task

As all WMC scores are based on the same input information–whether a certain to-be-remembered item was correctly recalled or not–correlations among them are inevitably high [7]. In that sense, correlations between PSC/ PWS and PNS for all tasks in both analysed (sub)samples are almost perfect (all *r*s = .99); the same is the case with ASC/ AWS and ANS (the smallest *r* = .98; S5 Table).

Correlations that combine partial with absolute measures are lower, but still very high: they range from *r* = .88 (correlations that SSPAN PSC/ PWS and PNS had with ASC/ AWS) to *r* = .93 (correlations between OSPAN PNS and ANS; S5 Table).

These correlations correspond to the values in Conway et al. [7]. On the basis of such results, the authors concluded that once the choice between partial and absolute scoring is made, non-weighted and weighted procedures become parallel. Still, keeping in mind that the correlations between partial and absolute scores are lower than perfect, the selection of one of these scoring procedures means taking other parameters into consideration as well. This issue will be further discussed in our concluding remarks.

It is again shown that abandoning the accuracy criterion on the processing component does not give rise to differences in observed values: the range of correlations between the different scores of one span task is the same regardless of whether the accuracy criterion was applied or not.

### Convergent validity

**Fig 1:**
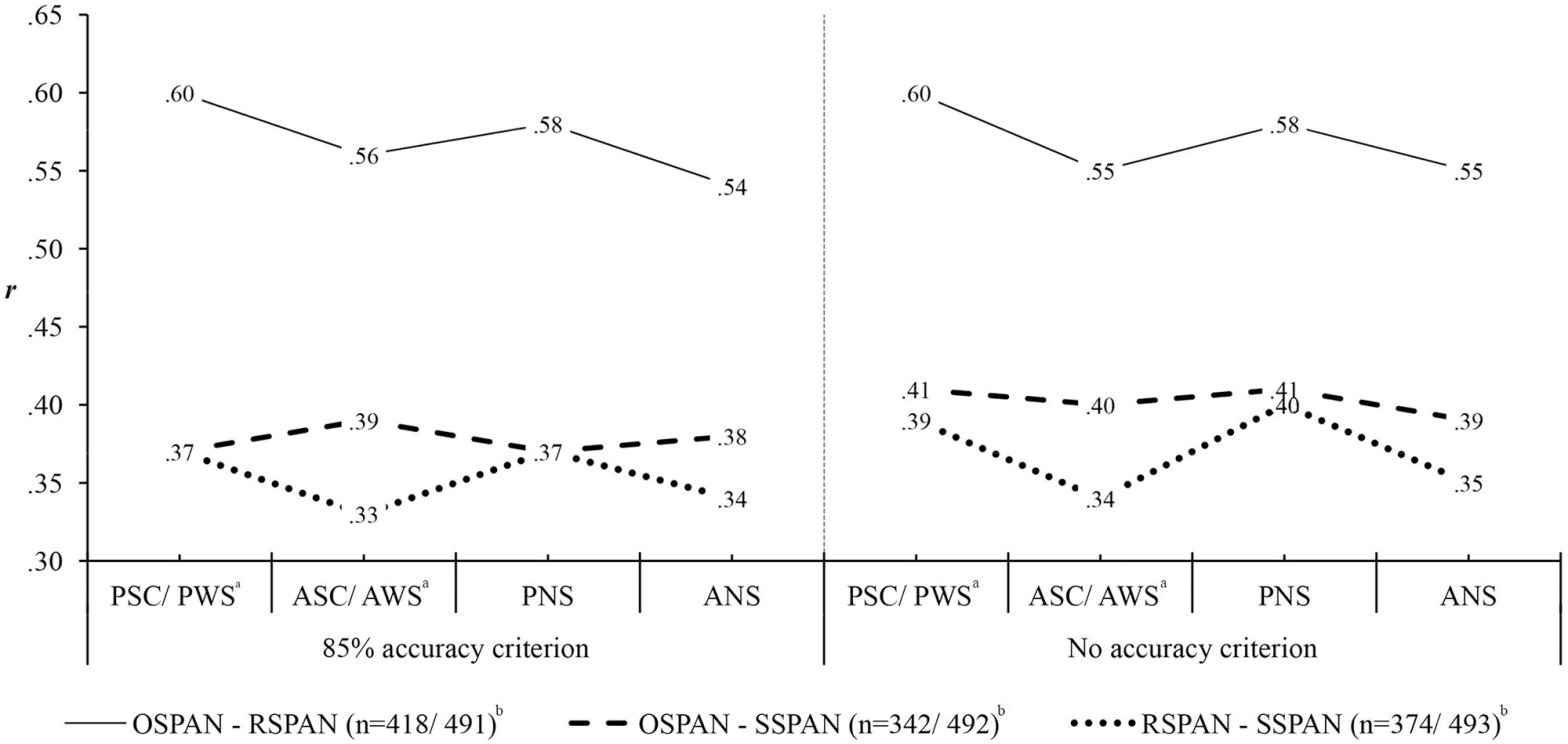
Correlations among equivalent working memory capacity (WMC) scores of different working memory (WM) span tasks according to the accuracy criterion on the processing component of the tasks. All *p*s <.001. OSPAN = operation span; RSPAN = reading span; SSPAN = symmetry span; PSC = partial score; ASC = absolute score; PNS = partial non-weighted score; ANS = absolute non-weighted score; PWS = partial weighted score; AWS = absolute weighted score. ^a^As PWS is a simple linear transformation of PSC, their intercorrelation is maximal (*r* = 1), and their correlations with other measures are identical. The same holds for AWS and ASC. ^b^Size of the subsample of participants above the 85% accuracy criterion/ size of the full sample with no accuracy criterion.

Fig 1 presents correlations between equivalent measures of different span tasks. For the 85% accuracy criterion, correlations were calculated in the subsample of participants who achieved the criterion on the processing component of both correlated tasks. All correlations are moderate to high and all are significant at the *p* <.001 level.

Correlations between the equivalent measures of OSPAN and RSPAN range from *r* = .54 (between ANSs in the subsample with an 85% accuracy criterion) to *r* = .60 (between PSCs/ PWSs, regardless of the accuracy criterion).

Correlations of SSPAN scores with their equivalents on OSPAN and RSPAN are generally lower. Thus, correlations between measures of SSPAN and OSPAN range from *r* = .37 (correlations between all types of partial scores in the subsample with an 85% accuracy criterion) to *r* = .41 (between all types of partial scores with no accuracy criterion). The lowest correlation between the equivalent measures of SSPAN and RSPAN is *r* = .33 (between ASC/ AWS in the subsample with 85% criterion), and the highest is *r* = .40 (between PNS with no accuracy criterion).

The range of correlation values and their pattern (stronger correlations between the non-spatial tasks) corresponds to findings in the literature (e.g. [8,21,27–30]). Also, correlations between partial scores are generally somewhat higher than correlations between absolute scores (see also [8]); the exception being correlations between OSPAN and SSPAN in the subsample with an 85% accuracy criterion, which increase slightly with absolute scores.

In general, such moderate to high correlations between different span tasks indicate that they indeed measure the same construct, but not in the same way [7].

Correlation values calculated with and without the accuracy criterion are, again, similar: the only correlation coefficient that decreased (from .56 to .55) with the omission of the accuracy criterion on the processing component of the span tasks was the one between ASC/ AWS of OSPAN and RSPAN.

### Criterion-related validity

The criterion validity of the WMC scores was estimated in relation to the *g*F measures. Three intelligence tests were applied in the study, the content of which approximate the stimulus material utilized in span tasks. Thus, NGAT demands arithmetic operations–analogous to OSPAN; VGAT implies verbal reasoning–similar to RSPAN; and Raven contains a certain spatial component–following the model of the dominantly spatial SSPAN (see [17]).

A composite *g*F measure for each participant was formed by averaging the z-scores accomplished on the three intelligence tests. Descriptive indicators and *α*s for intelligence measures are presented in S7 Table. These values, as well as all the following results, were obtained in the subsample of participants who completed all three intelligence tests and, in the case of analysis with the 85% accuracy criterion, achieved this criterion on the processing component on all three span tasks. (Sample size differences between the previous and the following analyses are due to missing data for participants who did not attend one or both intelligence test sessions.)

#### Correlations between working memory capacity (WMC) and fluid intelligence (*g*F)—Task level

**Fig 2:**
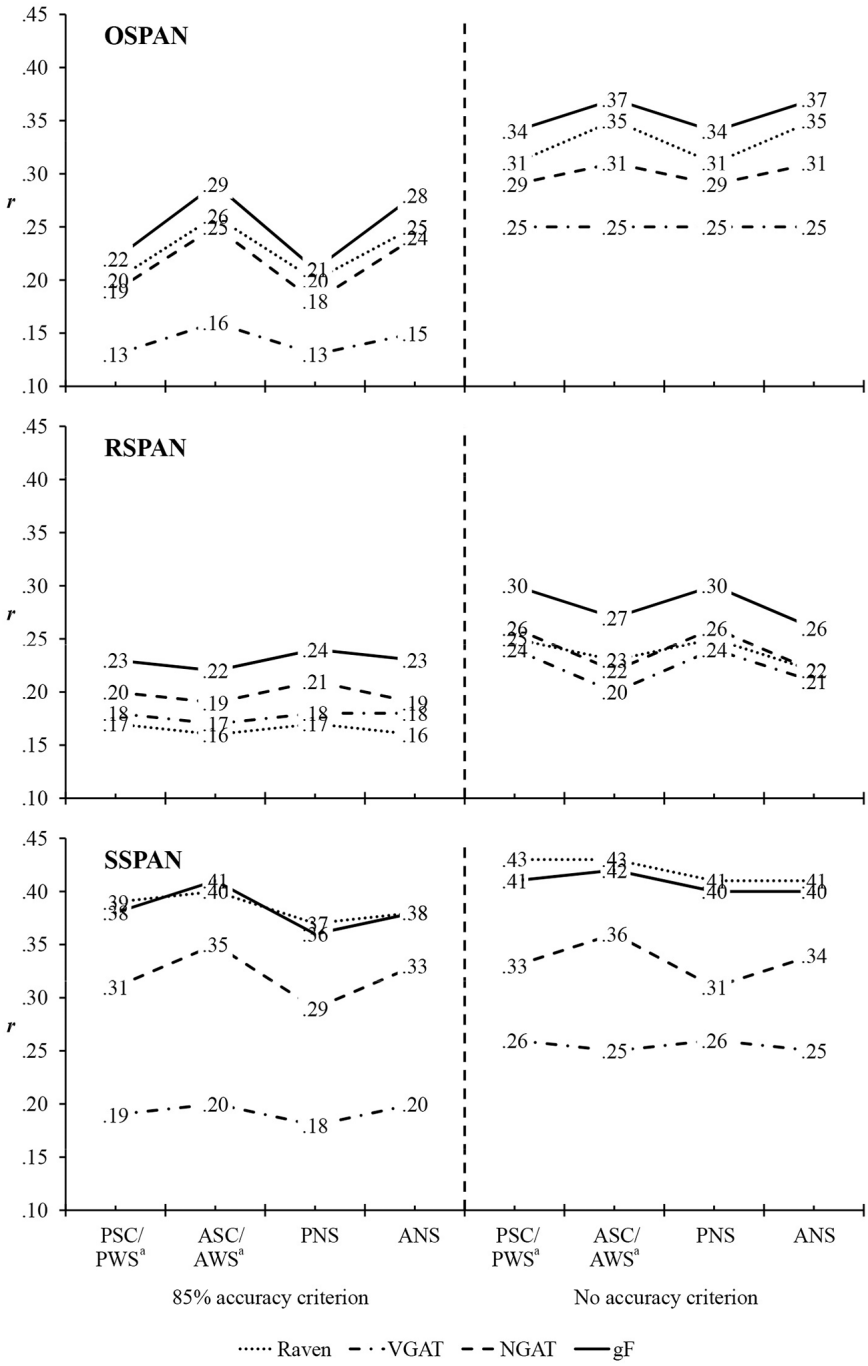
Correlations among working memory capacity (WMC) scores and measures of fluid intelligence (*g*F) according to the accuracy criterion on the processing component of the working memory (WM) span tasks. Size of the subsample with the 85% accuracy criterion: *n* = 311; size of the subsample with no accuracy criterion: *n* = 447. All correlations are significant: correlations below .15 are significant at the *p* <.05 level; correlations between .15 and .18 are significant at the *p* <.01 level; correlations above .18 are significant at the *p* <.001 level. OSPAN = operation span; RSPAN = reading span; SSPAN = symmetry span; PSC = partial score; ASC = absolute score; PNS = partial non-weighted score; ANS = absolute non-weighted score; PWS = partial weighted score; AWS = absolute weighted score; Raven = Raven Advanced Progressive Matrices; VGAT = Verbal General Ability Test; NGAT = Numerical General Ability Test. ^a^As PWS is a simple linear transformation of PSC, their intercorrelation is maximal (*r* = 1), and their correlations with other measures are identical. The same holds for AWS and ASC.

Fig 2 presents correlations between WMC scores and measures of *g*F for subsamples with and without 85% accuracy criterion on the processing component of the span tasks (the corresponding 95% confidence intervals for the correlations are presented in S8 Table).

Correlations between individual WMC scores and individual intelligence tests range from small to moderate and all are significant (at the *p* <.05 level or above). The smallest correlation (*r* = .13) was observed between OSPAN partial scores and VGAT in the subsample with the 85% accuracy criterion; the highest coefficients (*r* = .43) were those between SSPAN PSC/ PWS as well as ASC/ AWS and Raven, in the subsample with no accuracy criterion.

Correlations with individual intelligence tests, averaged across all scores of the individual span task at a given accuracy criterion, range from *r* = .14 (OSPAN–VGAT, the subsample with the 85% accuracy criterion) to *r* = .42 (SSPAN–Raven, the subsample with no accuracy criterion; S8 Table).

For individual span tasks, averaged correlations of the partial WMC scores with individual intelligence tests range from *r* = .13 (OSPAN–VGAT, the subsample with an 85% accuracy criterion) to *r* = .42 (SSPAN–Raven, the subsample with no accuracy criterion). For absolute WMC scores, that range is from *r* = .16 (OSPAN–VGAT and RSPAN–Raven, both in the subsample with 85% criterion) to *r* = .42 (for SSPAN–Raven, the subsample with no accuracy criterion; S8 Table).

WMC scores had somewhat stronger correlations with the *g*F composite measure (all *r*s are significant at *p* <.001 level) than with individual intelligence tests (the exception being SSPAN, which has slightly higher correlations with Raven than with the *g*F). The weakest correlation (*r* = .21) with the *g*F was for OSPAN PNS in the subsample with the 85% accuracy criterion. The highest correlation (*r* = .42) with the *g*F had SSPAN ASC/ AWS in the subsample with no accuracy criterion.

Correlations with the *g*F, averaged across all scores of the individual span task in a given subsample, range from *r* = .23 (for RSPAN in the 85% accuracy criterion subsample) to *r* = .41 (for SSPAN in the subsample with no accuracy criterion; S8 Table).

Partial and absolute WMC scores have the same range of average correlations with the *g*F composite: from *r* = .22 (for OSPAN in the case of partial scores and for RSPAN in the case of absolute scores, both in the 85% accuracy criterion subsample) to *r* = .41 (for SSPAN in the no accuracy criterion subsample, equally for partial and absolute scores).

These correlations correspond to the range of the correlation values in the literature (e.g. [9,19–21,27–29,31,32]).

Comparison of correlation values across fluid intelligence (*g*F) measures, span tasks, and working memory capacity (WMC) scores. Next, we considered whether the correlation coefficients between WMC and intelligence change significantly depending on the type of (a) intelligence test, (b) span task or (c) WMC score.

To check whether intelligence tests diverge on their correlations with span tasks, we tested for significant differences between correlations of the different intelligence tests (e.g. Raven vs. VGAT) with individual WMC scores (e.g. PSC OSPAN). These comparisons, as well as the following ones, were based on formula (7) in Steiger [33] for *t-test* of the difference between two dependent correlations with one variable in common (see also [22]). As this analysis for each span score included calculating an omnibus of three *t-tests*, in order to protect against Type I error a Bonferroni correction by a factor of 3 was applied, to adjust the alpha level (corrected alpha levels: .05/3 = .017 and .01/3 = .003). Here, only the significant differences will be summarized. The results of all *t-tests* conducted, and the corresponding *p* values, are presented in S9 Table.

All three intelligence tests have similar correlations with RSPAN. With OSPAN and SSPAN, in general, Raven correlated the strongest, NGAT somewhat less, and VGAT the weakest. As regards significant differences, these occurred only for SSPAN. All SSPAN scores correlated significantly higher with Raven than with VGAT in both subsamples. In addition, in the subsample with no accuracy criterion, all types of SSPAN partial scores correlated higher with Raven than with NGAT. Also, SSPAN ASCs/ AWSs in both subsamples had higher correlations with NGAT than with VGAT.

In order to test whether one span task, compared to the other two, tends to correlate more strongly to *g*F measures, we tested for statistical differences between correlations of equivalent scores of two span tasks (e.g. PSC OSPAN vs. PSC RSPAN) with the same intelligence test (e.g. Raven). Since this analysis also included an omnibus of three *t-tests* per span score, the Bonferroni correction of the alpha level by a factor of 3 was applied again (with *p* values between .024 and .018 considered as indicators of marginal differences).

Comparison of correlations of different span tasks with the same intelligence test shows that SSPAN correlates with Raven significantly higher than both remaining span tasks; the exceptions are OSPAN ANS in the subsample with an 85% accuracy criterion as well as OSPAN ASC/ AWS, PNS, and ANS in the subsample with no accuracy criterion, which all correlate with Raven as strong as the equivalent SSPAN scores. SSPAN absolute scores also have (marginally) higher correlations with NGAT, compared to equivalent RSPAN scores. Additionally, in the subsample with no accuracy criterion, all types of OSPAN absolute scores have significantly higher zero-order correlations with Raven than corresponding RSPAN scores. All three span tasks have comparable correlations with VGAT.

Compared with OSPAN and RSPAN, SSPAN generally has stronger correlations with the *g*F composite as well. In the 85% accuracy criterion subsample, all SSPAN scores correlate significantly higher with *g*F than the corresponding scores of both remaining tasks, the exceptions being OSPAN absolute scores as well as RSPAN PNS. In the subsample with no accuracy criterion, correlations of all SSPAN absolute scores with *g*F are higher than those of RSPAN scores; a marginal difference is observed for PSC/ PWS. Also in this subsample, all types of OSPAN absolute scores have (marginally) higher correlations with *g*F composite than corresponding RSPAN scores.

Previous studies have shown a tendency for partial WMC scores to have stronger correlations with the *g*F measures, compared with the absolute WMC scores [22,34]. In this regard, our study does not give a clear answer. Namely, we tested for statistical differences between correlations of analogous partial and absolute scores of the same span task (e.g. PSC OSPAN vs. ASC OSPAN) with the same intelligence test (e.g. Raven). This time the Bonferroni correction of the alpha level was implemented with a factor of 4, since the analysis included an omnibus of four *t-tests* per span task (the corrected alpha levels: .05/4 = .013 and .01/4 = .003; this time, indicators of marginal differences were considered to be *p* values between .024 and .014).

The results of the *t-tests* conducted demonstrate that correlations of partial and absolute WMC scores with the intelligence measures do not differ in general. The only exceptions were observed in the case of OSPAN: compared to analogous partial scores, all types of OSPAN absolute scores in the subsample with the 85% accuracy criterion as well as ANS in the subsample with no accuracy criterion had (marginally) significantly higher correlations with Raven; this was also the case for the correlations of all OSPAN absolute scores with NGAT and *g*F composite in the subsample with an 85% accuracy criterion.

Finally, when excluding the accuracy criterion, correlations between WMC and intelligence measures only become larger, not smaller (the observed increases in the values of the correlation range between 1 and 13 points). This increase of correlations is due to the increase of variability of both the WMC and *g*F measures that occurs once the results of the participants under the accuracy criterion were included in the analysis. These originally excluded cases mainly fall under the arithmetic means of both the WMC and *g*F variables. Thus, omitting the accuracy criteria leads to a widening of the sample to include mainly lower results, in the case both of WMC and *g*F. (As an illustration of which cases enter the analysis when omitting the accuracy criterion, please see the scatter plots of correlations between PCSs of all three span tasks and *g*F measures, presented in S1 Fig.) For a similar conclusion on the effect of decrease of the mean general ability of the sample on the increase of correlations among span tasks, see [8], pp. 169.

#### Correlations between working memory capacity (WMC) and fluid intelligence (*g*F)—Latent level

In order to explore the relationship between WMC and *g*F more thoroughly, all measures of WMC and intelligence were input into a confirmatory factor analysis. In both the subsamples analysed, a separate confirmatory factor analysis was conducted for each type of WMC score.

**Fig 3:**
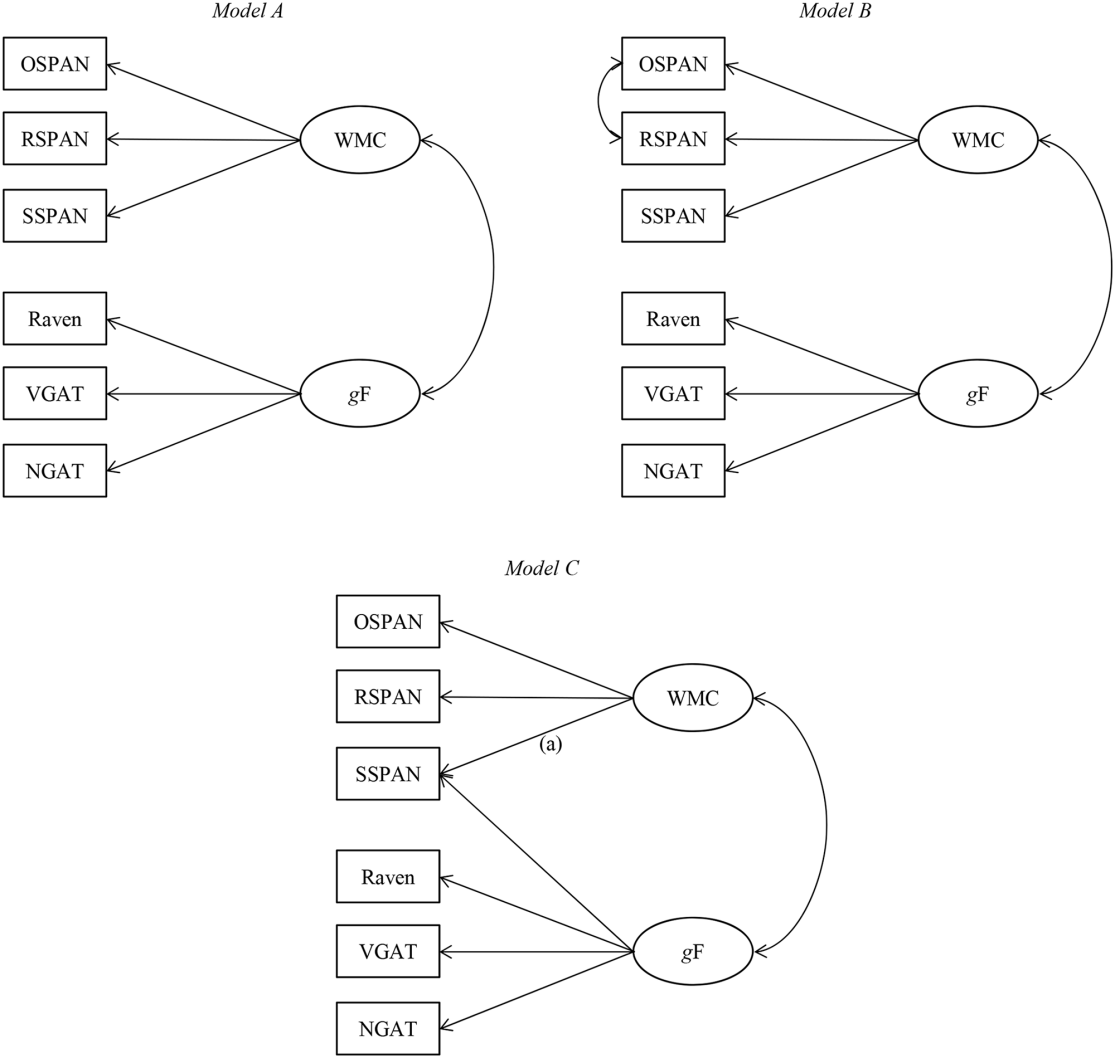
Schematic presentation of path models for the structural equation analysis of the relation between working memory capacity (WMC) and fluid intelligence (*g*F). Each model was tested for each type of WMC score and in subsamples with and without the 85% accuracy criterion on the processing component of span tasks. The paths leading from the constructs (circles) to the manifest variables (rectangles) represent the loadings of each measure on that construct. The double-headed paths between the WMC factor and the *g*F factor represent the correlation between the two constructs. The double-headed path between the OSPAN and RSPAN manifest variables in Model B represents the correlation between the residuals of these two measures. By exclusion of path (a) in Model C it was possible to test an additional model in which SSPAN is related only to the *g*F latent factor, but not with WMC factor. All paths and loadings in all models are significant at the *p* <.001 confidence level, for each type of WMC scores and in all analysed subsamples. OSPAN = operation span; RSPAN = reading span; SSPAN = symmetry span; Raven = Raven Advanced Progressive Matrices; VGAT = Verbal General Ability Test; NGAT = Numerical General Ability Test.

In the initial analysis (Model A in Fig 3), we defined two latent factors: the WMC factor, consisting of three span tasks, and the *g*F factor, based on three intelligence tests. The goal of the analysis was to examine if our results would replicate this simple solution which was specified in concordance with models in the literature. Specifically, we predicted: (a) the extraction of two univocal and distinct latent factors–WMC and *g*F; and (b) the correlation between these two factors to be around .60, the magnitude reported in previous studies [9,15,17].

However, Model A did not fit the observed data, and this was the case for all types of WMC scores in both subsamples. All the resulting chi-square goodness of fit tests were significant, indicating that the observed data did not fit the model (S10 Table; two additional fit indices for Model A, not presented in S10 Table, were indicative of its poor fit for all types of WMC scores and in both subsamples [26]: χ^2^/*df* >2 and NNFI≤.90).

In order to improve the fit of the model, in a subsequent analysis we specified a more liberal Model B (Fig 3) that allows for correlations between the task-specific error terms of OSPAN and RSPAN. These correlated residuals represent the variance that is common to these two span tasks, but not SSPAN [17]. In accordance with Kane et al. [17], inclusion of the correlated residuals of OSPAN and RSPAN into the model was based on three criteria. First, the correlation between the OSPAN and RSPAN residuals is theoretically justified. Namely, compared to SSPAN, these two span tasks have the following similarities: (a) the to-be-remembered items in OSPAN and RSPAN are of the same, verbal modality (individual letters), in contrast to spatial positions of squares in SSPAN; (b) the recall screen, i.e. the manner of reproducing the to-be-remembered items in OSPAN and RSPAN is identical; (c) in accordance with their similar structure, the instructions for OSPAN and RSPAN are more similar compared to those used for SSPAN; (d) OSPAN and RSPAN are of equal length with a total of 75 to-be-remembered items, contrasting with 42 in SSPAN. The second criterion was the statistical significance of the correlation between residuals, and that was the case for all types of OSPAN and RSPAN scores in both subsamples (S10 Table). Finally, owing to the correlated OSPAN and RSPAN residuals, the fit for Model B compared to the fit for Model A was significantly improved according to a chi-square difference test (at the *p* <.001 level), and this was the case for all types of WMC scores, again–regardless of the accuracy criterion (S10 Table). The fit for Model B, for all WMC scores and in both subsamples, was very good (S10 Table): all chi-square goodness of fit tests were insignificant at the *p*>.05 level; all root-mean-square errors of approximation (RMSEA) and all standardized root-mean-square residuals (SRMR) values were <.05; all normed fit index (NFI), all non-normed fit index (NNFI), and all confirmatory fit index (CFI) values were >.95 [26]. In accordance with expectations, Model B confirms the existence of univocal and distinct latent factors of WMC and *g*F, and also confirms the expected magnitude of correlation between the two–the lowest correlation of .56 was observed in the model based on PNS in the subsample with the 85% accuracy criterion; the highest correlation of .71 was observed in the model with ASC/ AWS in the subsample with no accuracy criterion. It is also noticeable that SSPAN has higher loadings on WMC factor than both OSPAN and RSPAN.

Versions of Model B that include absolute WMC scores, compared to those based on partial scores, nominally generate higher correlations between the constructs of WMC and *g*F. However, judging by the overlap between the 95% confidence intervals of the WMC-*g*F correlation coefficients, models with absolute and partial scores do not significantly differ in this regard (S10 Table).

By omitting the accuracy criterion on the processing component of the span tasks, indicators of fit for Model B remain stable. Also, correlations between latent variables of WMC and *g*F do not decrease; moreover, these correlations have somewhat higher values (from 6 to 11 points) in the subsample with no accuracy criterion (S10 Table).

However, the previously observed pattern in which SSPAN correlates with the two remaining WMC span tasks in a similar way as it correlates with the *g*F measures suggested that we should test one more model, which in this case was *post hoc*. In this model, SSPAN is correlated both with WMC and *g*F factor (Model C, Fig 3). Model C became equivalent to Model B, i.e. the fit indices for these two models became identical. Furthermore, SSPAN has very similar loadings on WMC and *g*F factors in Model C (S10 Table). Therefore, Model C confounds initial expectations in the sense that it does not confirm the univocal factors of WMC and *g*F. Thus, the correlation between these factors, depending on the type of the WMC score and on the application of the accuracy criterion, drops to between .34 and .51 (S10 Table).

In considering which of these two models is more acceptable (B or C), we were guided by the *a priori* theoretical postulates according to which WMC and intelligence are different, but strongly related constructs (see [16,35,36]), as well as by the fact that SSPAN has been proven to be a valid WMC measure [8]. Model B, unlike Model C, complies to these principles and has been additionally confirmed by numerous findings in the literature (e.g. [15,17,28,29,36]). On the other hand, an alternative explanation according to which SSPAN is as good an indicator of intelligence as it is a measure of WMC is made implausible by the lack of fit of the model in which this span task is correlated only with the *g*F latent factor, but not with the WMC factor. This is the model specified when relation (a) is excluded from Model C (Fig 3). For this model, for all types of WMC scores in both subsamples, statistically significant chi-square goodness of fit tests (at the *p* <.05 level) indicate a deviation from empirical data (S10 Table). Also, no arguments have been found to confirm the existence of two separate WMC factors: verbal (based on OSPAN and RSPAN) and spatial (based on SSPAN). The exploratory factor analysis with OSPAN, RSPAN and SSPAN prefers a one-factor as opposed to a two-factor solution, regardless of the type of span scores and the accuracy criterion applied on the processing component of span tasks (S11 Table; for a more detailed discussion on the generality of WMC, please see [17,37]).

## General discussion

The results of the study show that the group administration of automated span tasks does not compromise their psychometric characteristics. All the tested tasks demonstrate an adequate internal consistency with *α* values equal to or above the commonly used criterion of .70 (see [7,8]); the only exception being all types of SSPAN absolute scores, with *α*s in the range of .60. Furthermore, all tasks have satisfactory convergent construct validity as well as criterion validity estimated in relation to the *g*F measures. The obtained psychometric indicators correspond to the norms established by the individual administration of span tasks [7–9].

The study seems to reveal a trend according to which the spatial SSPAN task deviates from the two non-spatial tasks, OSPAN and RSPAN. Primarily, in comparison to other two tasks, a higher percentage of participants failed to meet the 85% accuracy criterion on the SSPAN processing component. Internal consistency is lower for SSPAN. Also, the correlations of SSPAN with OSPAN and RSPAN are generally lower than the inter-correlations of these two non-spatial tasks. At the same time, SSPAN is more strongly related to measures of intelligence (Raven, NGAT and the *g*F composite measure). Also, it contributes the most to the WMC latent factor once the correlation of the residuals of OSPAN and RSPAN is included in the structural equation model. These results fit nicely into the ongoing debate on the consistently observed pattern that spatial measures of WMC, compared to verbal measures, are more strongly related to general cognition, e.g. *g*F [17] or executive attention [38]; this asymmetry in relation between verbal and spatial measures of WMC and higher-order cognition is yet to be fully integrated in the models of WM [39]. It seems that SSPAN, owing to its difficulty and the modality of the stimulus material, is more saturated with the fluid abilities factor and, hence, closer to *g*F. On the other hand, the less demanding OSPAN and RSPAN encompass the more crystallized abilities of arithmetic and reading, respectively. It is for that reason, and in spite the somewhat less adequate reliability of its absolute scores, that SSPAN is recommended as a valuable measure of WMC. In combination with the non-spatial span tasks, SSPAN (a) improves the construct validity of measuring WMC by encompassing the aspects of WMC that non-spatial tasks either do not measure or measure to a lesser extent; and, in a related way, (b) improves the sensitivity of measuring WMC by maximizing the variability of the results, thanks to the demands it puts in front of the participants. Thus, we recommend, whenever possible, to use several WMC measures in combination, especially measures based on different modalities–specifically, verbal and spatial. Conway et al. [7] give the same recommendation when they state that in the situation when a construct is measured by imperfect instruments, the best approach possible is to combine several reliable measures that do not overlap completely (see also [11]). Such multiple operationalization of constructs receives its full meaning in latent variable analysis, as our results also prove. Compared to the zero-order correlations between the manifest variables of WMC and *g*F, the correlations between the latent variables of WMC and *g*F reached considerably higher values. Accordingly, it is exactly the SSPAN scores that contribute the most to the WMC factor.

In considering which type of WMC scores should be preferred, the higher internal consistency and somewhat higher convergent validity (the exception being the relation between OSPAN and SSPAN) give preference to partial as opposed to absolute scoring procedures. For the same conclusion and additional discussion on the psychometric advantages of the partial scores, see [7,8].

In choosing between weighted and non-weighted WMC scores (within partial or absolute scoring procedures), the criteria for the decision were not so clear. Primarily, those are almost perfectly parallel measures. Thus, Conway et al. [7] leave the choice between the weighted and non-weighted procedures to the researchers, but emphasise that non-weighted scoring is more in accordance with standard psychometric procedures. Lalović and Vejnović [40] prefer non-weighted scoring more explicitly. In their view, there is no reason to believe that the same number of correctly recalled to-be-remembered items is a better indicator of WMC when the items belong to a longer, compared to a shorter set, especially because in span tasks the participants do not know the length of the set in advance.

In conclusion, the study does not identify a single argument for maintaining the 85% accuracy criterion on the processing component of span tasks for the purpose of filtering results for further analyses. In omitting the accuracy criteria, there were not any substantial changes in any of the analysed psychometric indicators of the tasks. Furthermore, negative correlations between the number of errors on the processing component and the number of to-be-remembered items were observed for all tasks, regardless of the accuracy criteria. In other words, participants who performed better on the processing component of span tasks achieved higher WMC scores. Such findings negate the possibility that participants invested all their processing resources into memorizing to-be-remembered items, ignoring thus the processing component of the tasks. For that reason, we recommend altering the instruction to the participants that they should maintain the 85% accuracy on the processing component of the span task into the instruction that they should focus on both the memory and processing component of the task equally as they are equally important. At the same time, we recommend that researchers omit the accuracy criterion when filtering the results for further statistical analysis. The consequent fall in the number of discarded results will directly improve the efficiency of administration of WM span tasks.

## Supporting information

S1 Table Structure of the (sub)samples according to the working memory (WM) span task and accuracy criterion on the processing component of the task. (TIF)

S2 Table Descriptive statistics for six working memory capacity (WMC) scores and three processing accuracy variables according to the working memory (WM) span task and accuracy criterion on the processing component of the task (85%/ 80%/ 75%/ 50%/ no accuracy criterion). (TIF)

S3 Table Correlations among working memory capacity (WMC) scores and processing errors according to the working memory (WM) span task and accuracy criterion on the processing component of the task (85%/ 80%/ 75%/ 50%/ no accuracy criterion). (TIF)

S4 Table Cronbach’s *α* for six working memory capacity (WMC) scores according to the working memory (WM) span task and accuracy criterion on the processing component of the task (85%/ 80%/ 75%/ 50%/ no accuracy criterion). (TIF)

S5 Table Correlations among different working memory capacity (WMC) scores of the same working memory (WM) span task according to the accuracy criterion on the processing component of the task (85%/ 80%/ 75%/ 50%/ no accuracy criterion). (TIF)

S6 Table Correlations among equivalent working memory capacity (WMC) scores of different working memory (WM) span tasks according to the accuracy criterion on the processing component of the tasks (85%/ 80%/ 75%/ 50%/ no accuracy criterion). (TIF)

S7 Table Descriptive statistics and *α*s for the measures of fluid intelligence (*g*F) according to the accuracy criterion on the processing component of the working memory (WM) span tasks. (TIF)

S8 Table Correlations among working memory capacity (WMC) scores and measures of fluid intelligence (*g*F) with accompanying 95% confidence intervals (CIs) according to the accuracy criterion on the processing component of the working memory (WM) span tasks. (TIF)

S9 Table *t-tests* and corresponding *p* values for the differences between two dependent correlations with one variable in common (a) Difference between correlations of different measures of fluid intelligence (*g*F) with the same working memory capacity (WMC) score; (b) Difference between correlations of equivalent scores of two working memory (WM) span tasks with the same measure of fluid intelligence (*g*F); (c) Differences between correlations of analogous partial and absolute scores of the individual working memory (WM) span task with the same measure of fluid intelligence (*g*F).
(TIF)

S10 Table Fit indices, factor loadings and correlations with accompanying 95% confidence intervals (CIs) for all models according to the type of working memory capacity (WMC) score and accuracy criterion on the processing component of the working memory (WM) span tasks. (TIF)

S11 Table Exploratory factor analysis (EFA) and total variance explained for the working memory (WM) span tasks according to the working memory capacity (WMC) score and accuracy criterion on the processing component of the task (85%/ 80%/ 75%/ 50%/ no accuracy criterion). (TIF)

S1 Fig Scatter plots of correlations among PCS and measures of intelligence according to the working memory (WM) span task and accuracy criterion on the processing component of the task. Panel (A): scatter plots for OSPAN and RSPAN. Panel (B): scatter plots for SSPAN.
(TIF)
