# Supplementary figures and images for: Working memory span tasks: Group administration and omitting accuracy criterion do not change metric characteristics
Source: PLoS One. 2018 Oct 11;13(10):e0205169. doi: 10.1371/journal.pone.0205169 (PMC6181321; doi:10.1371/journal.pone.0205169)

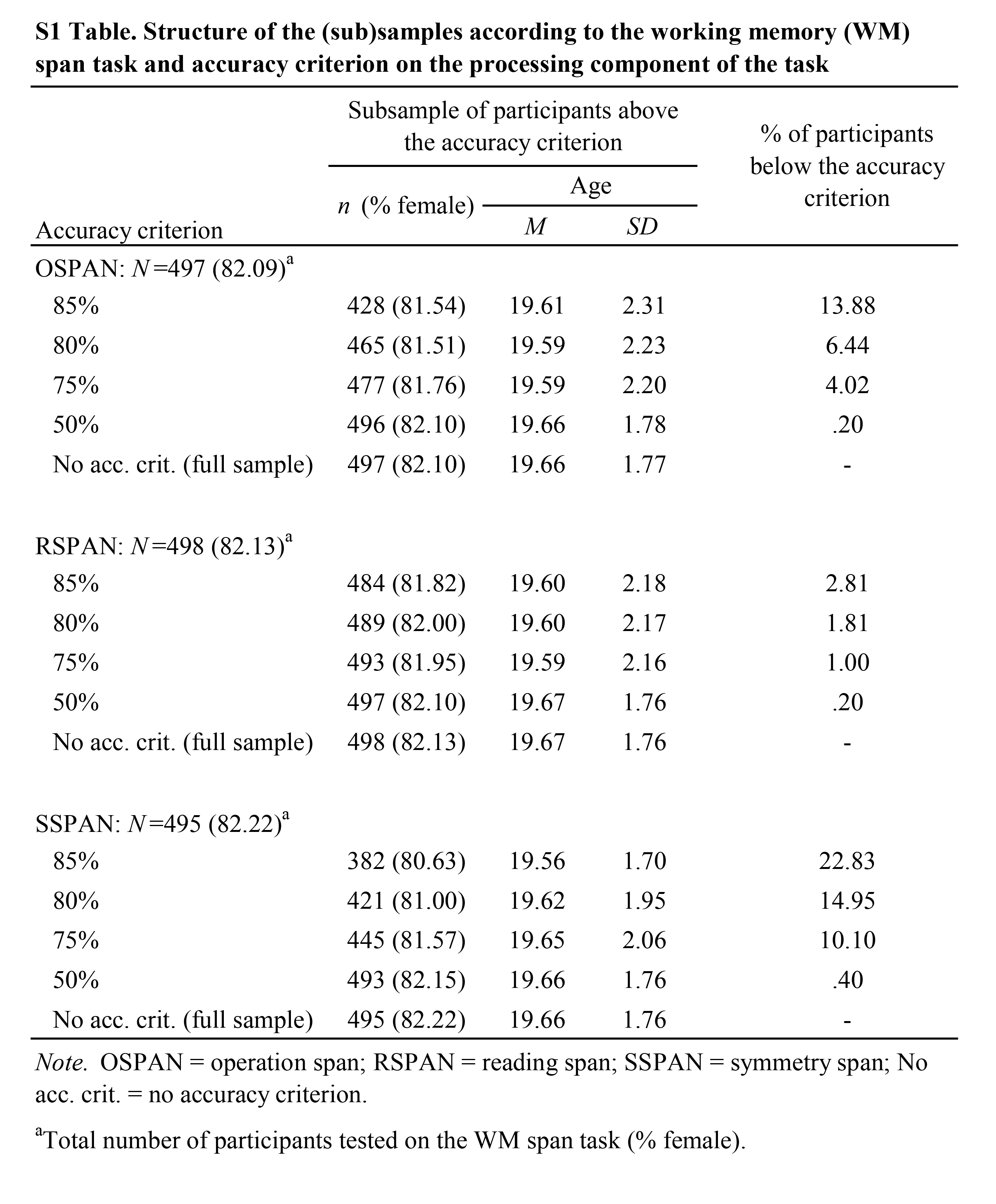

Supplement: S1 Table — (TIF) [file pone.0205169.s001.tif]

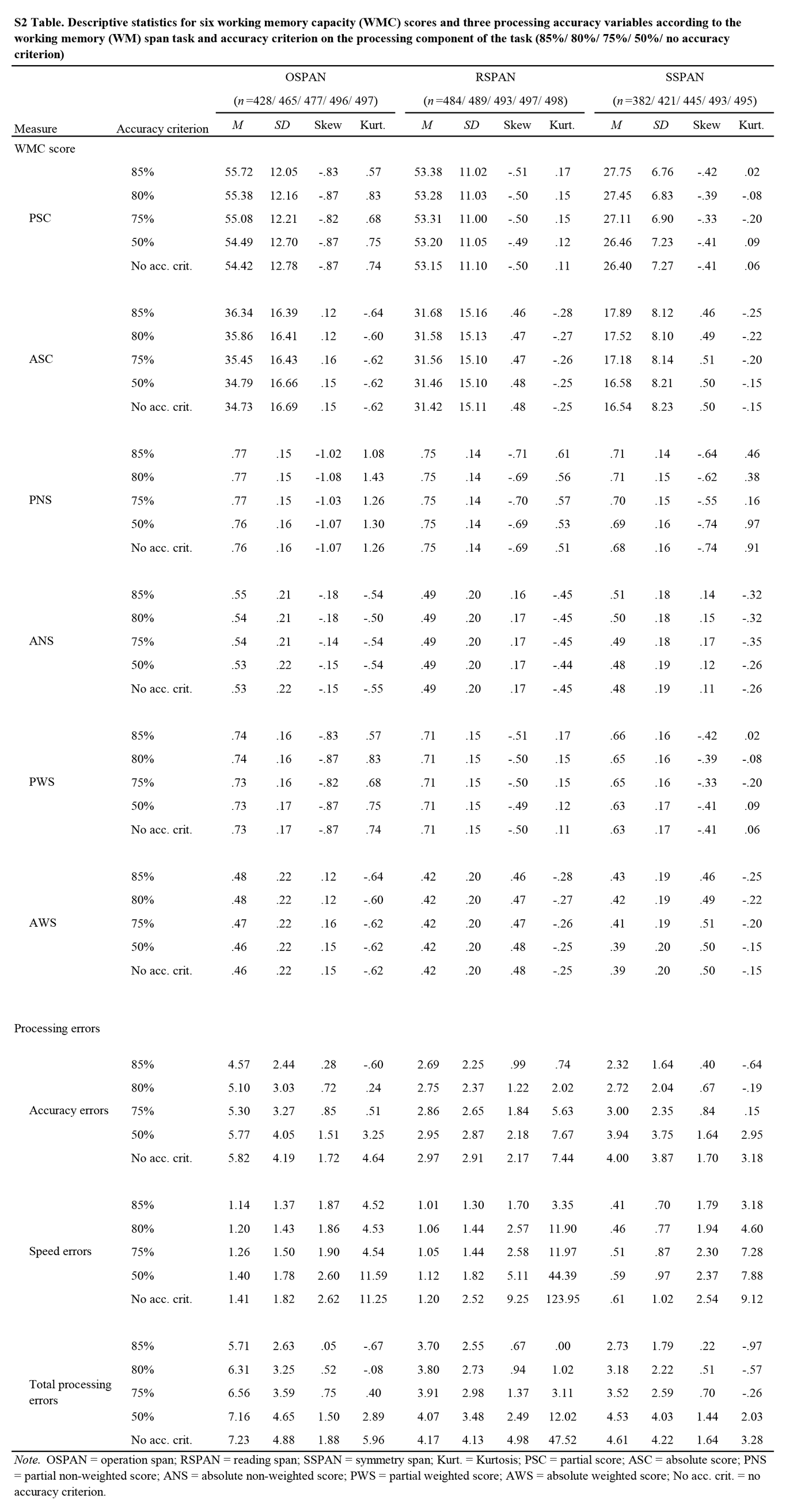

Supplement: S2 Table — (TIF) [file pone.0205169.s002.tif]

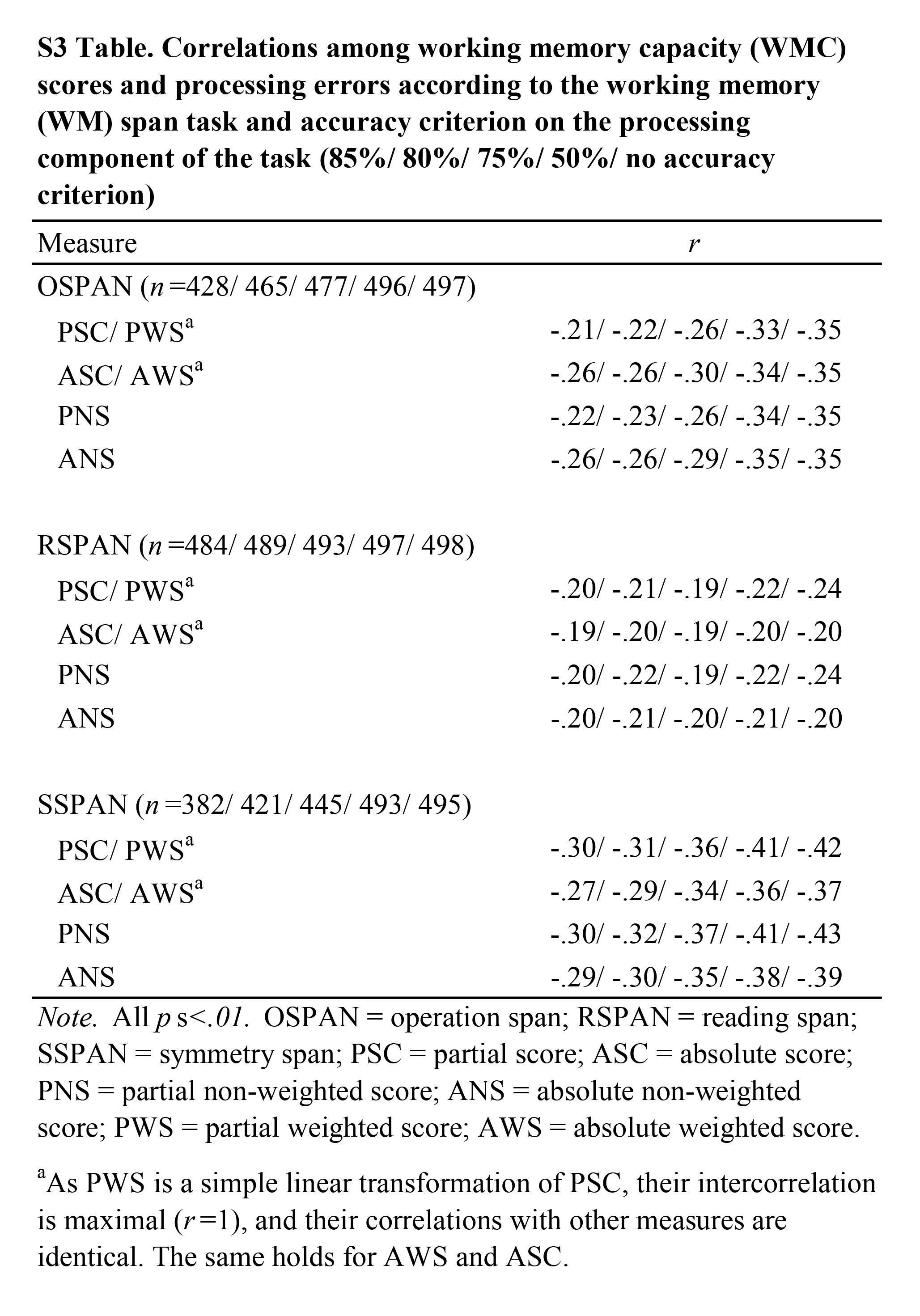

Supplement: S3 Table — (TIF) [file pone.0205169.s003.tif]

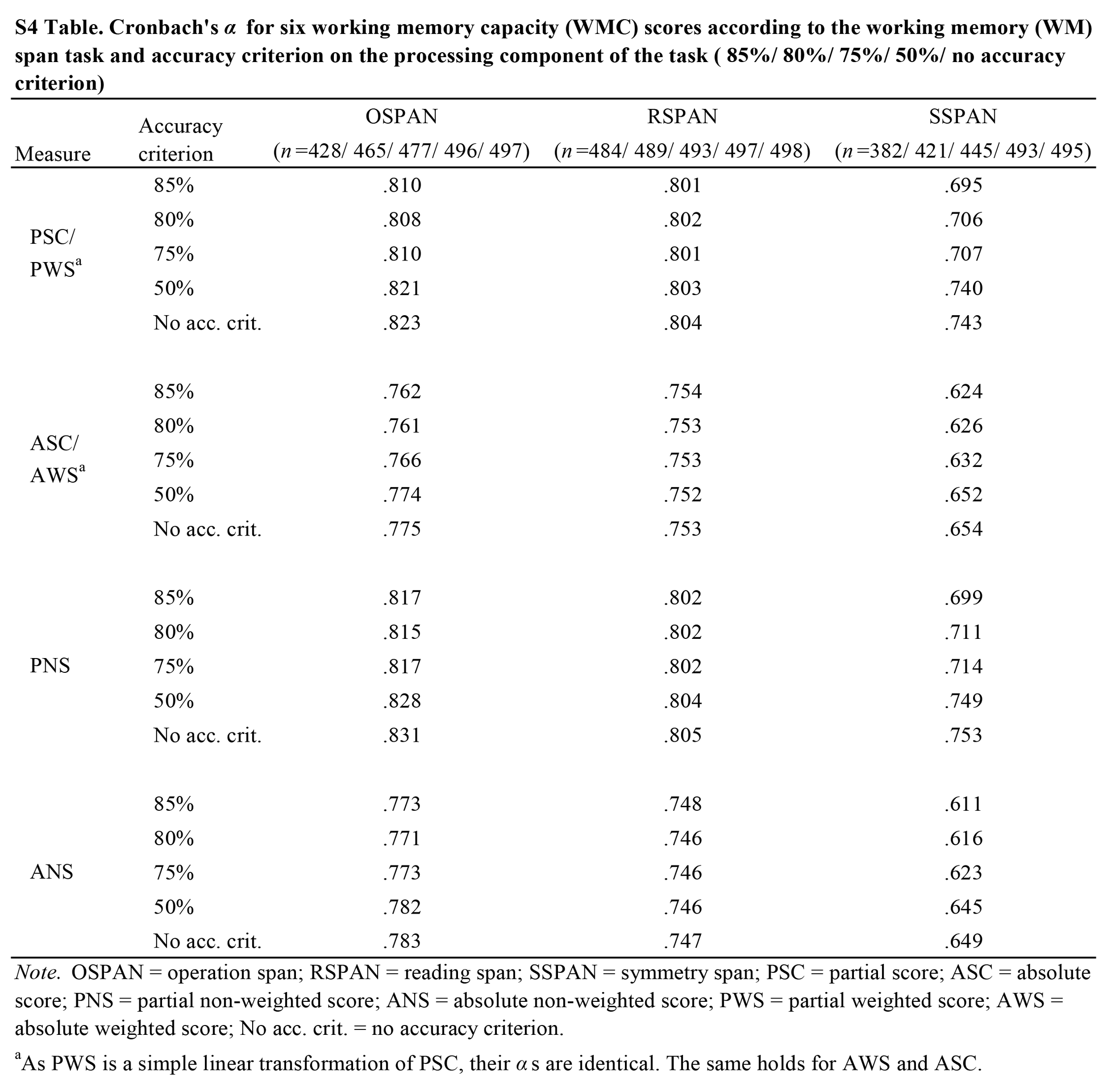

Supplement: S4 Table — (TIF) [file pone.0205169.s004.tif]

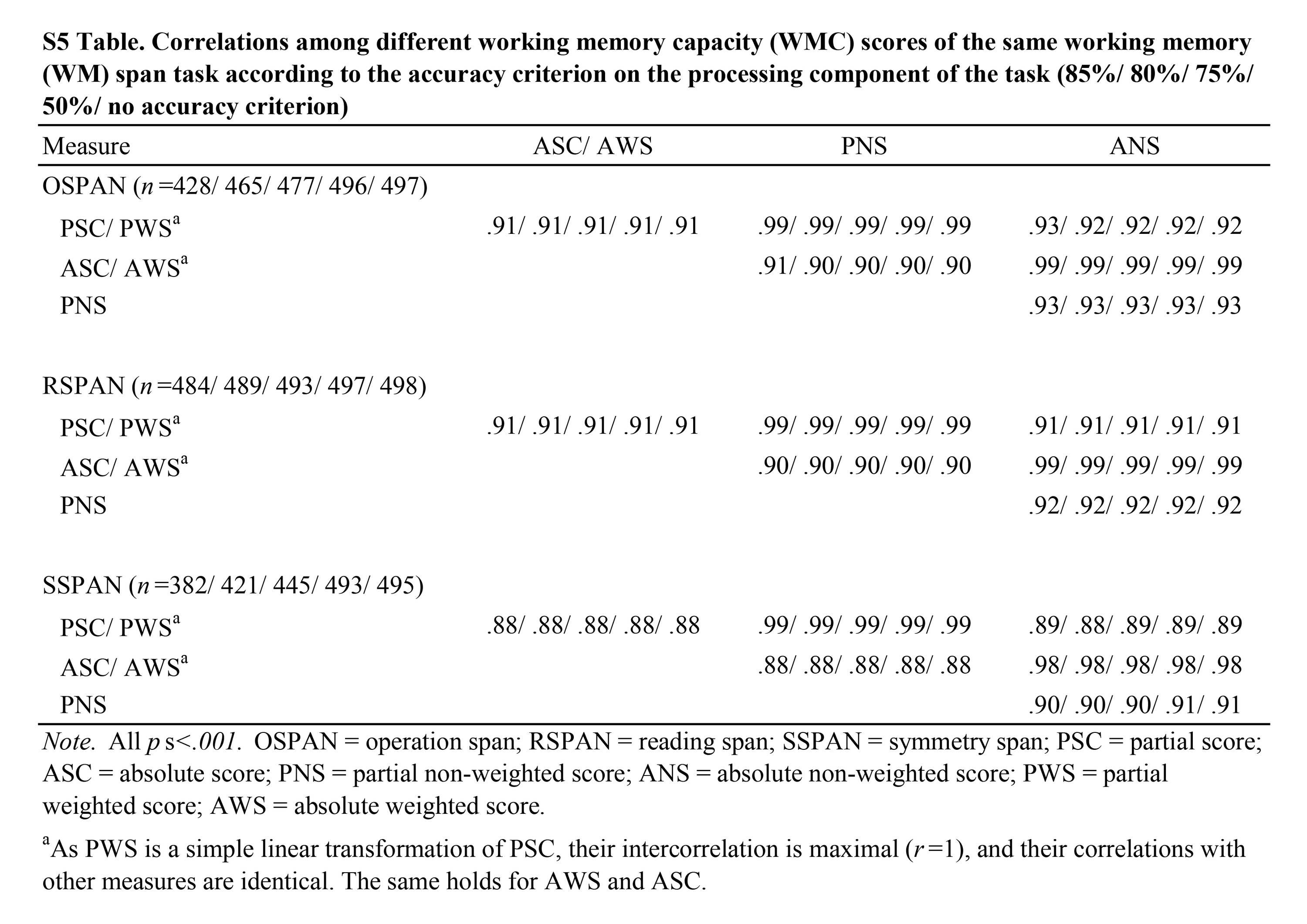

Supplement: S5 Table — (TIF) [file pone.0205169.s005.tif]

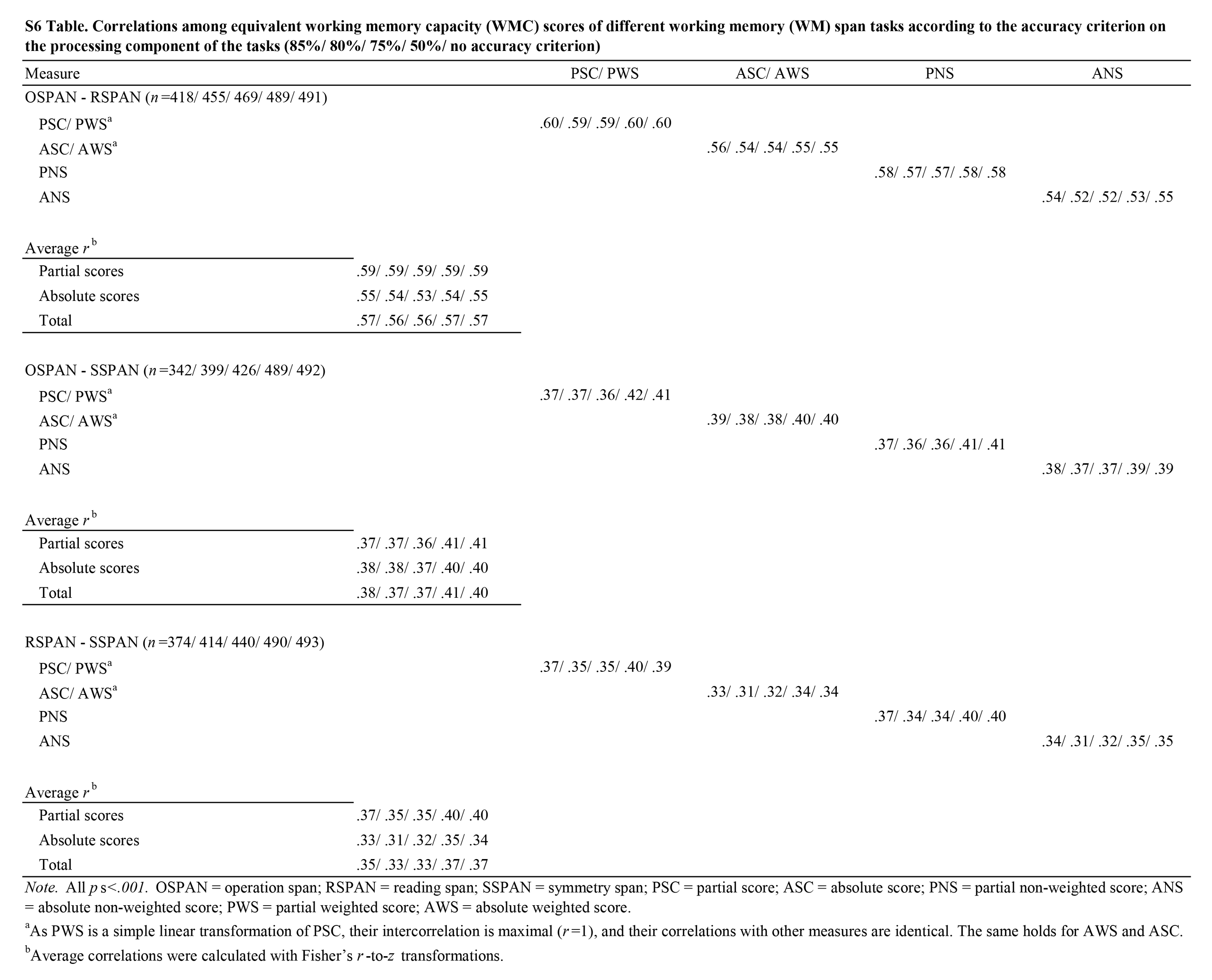

Supplement: S6 Table — (TIF) [file pone.0205169.s006.tif]

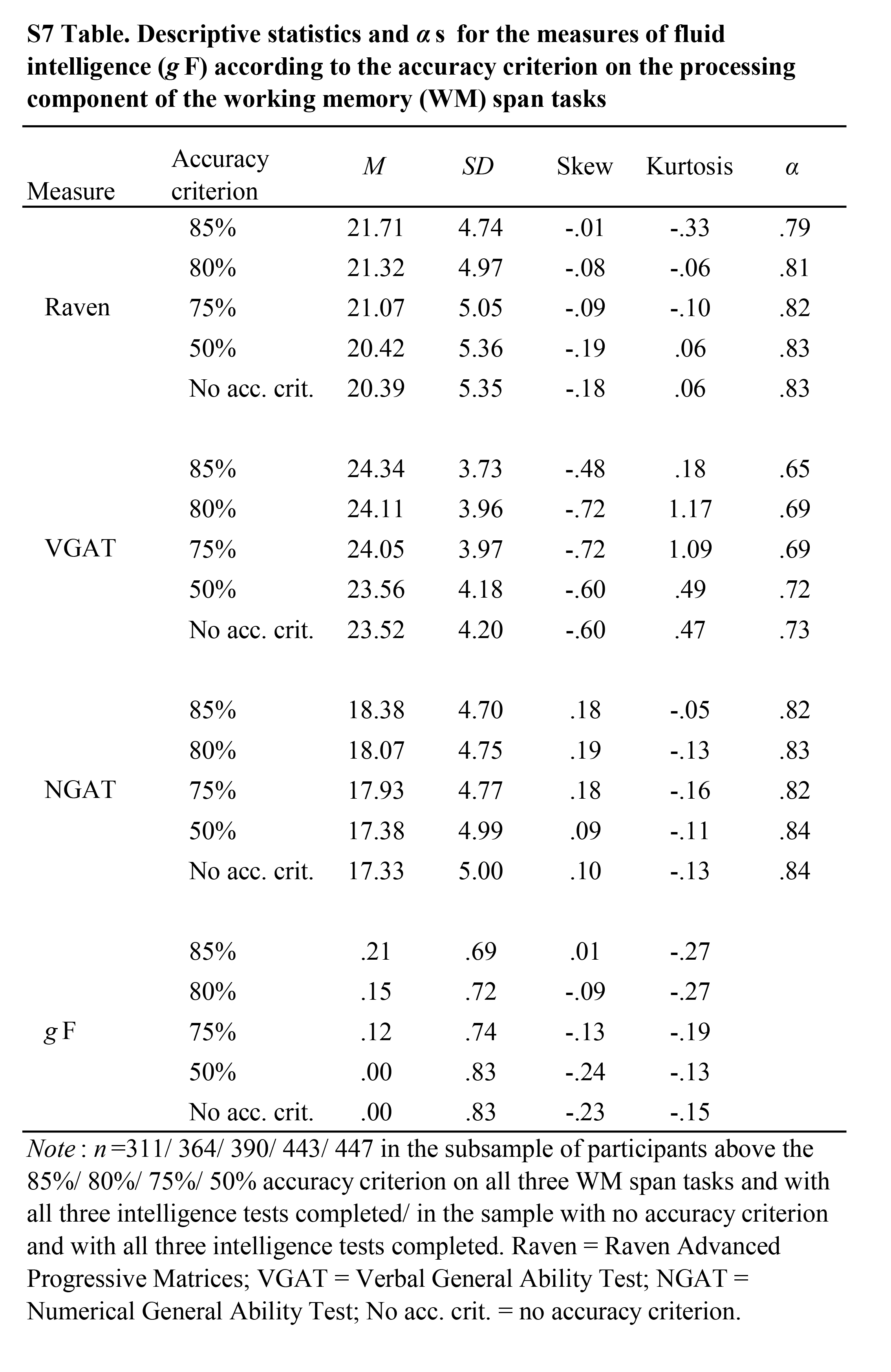

Supplement: S7 Table — (TIF) [file pone.0205169.s007.tif]

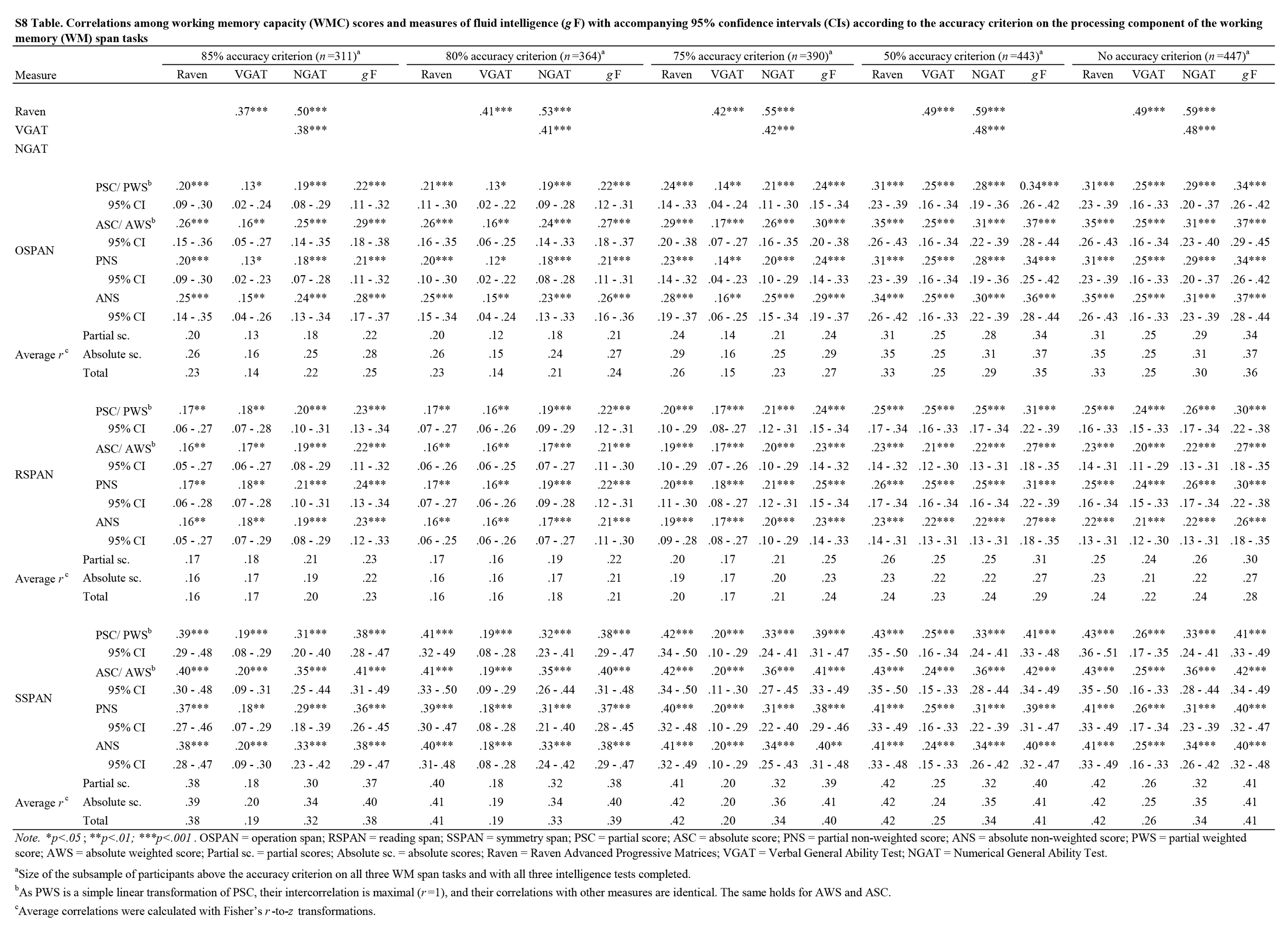

Supplement: S8 Table — (TIF) [file pone.0205169.s008.tif]

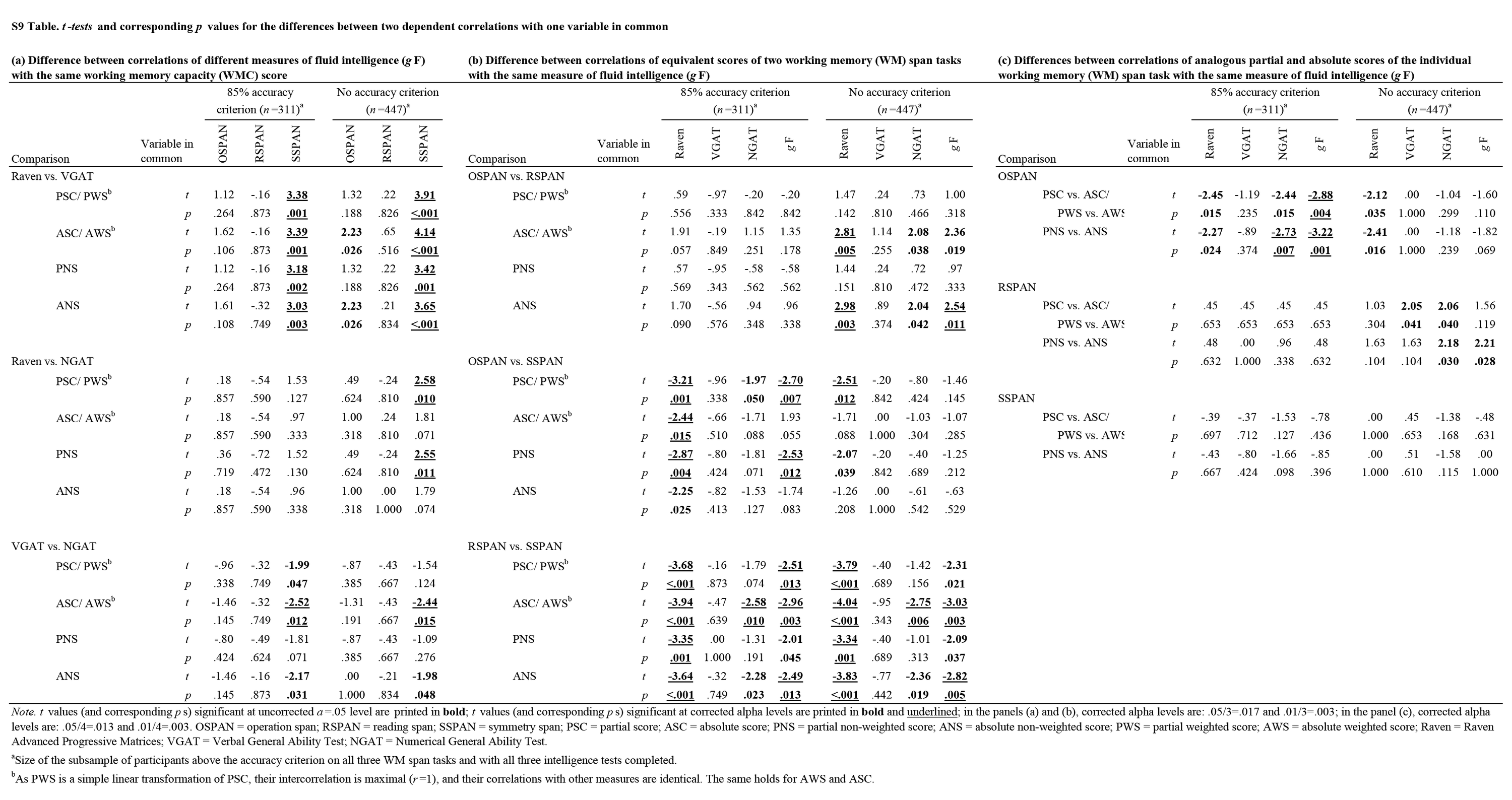

Supplement: S9 Table — t-tests and corresponding p values for the differences between two dependent correlations with one variable in common (a) Difference between correlations of different measures of fluid intelligence (gF) with the same working memory capacity (WMC) score; (b) Difference between correlations of equivalent scores of two working memory (WM) span tasks with the same measure of fluid intelligence (gF); (c) Differences between correlations of analogous partial and absolute scores of the individual working memory (WM) span task with the same measure of fluid intelligence (gF). (TIF) [file pone.0205169.s009.tif]

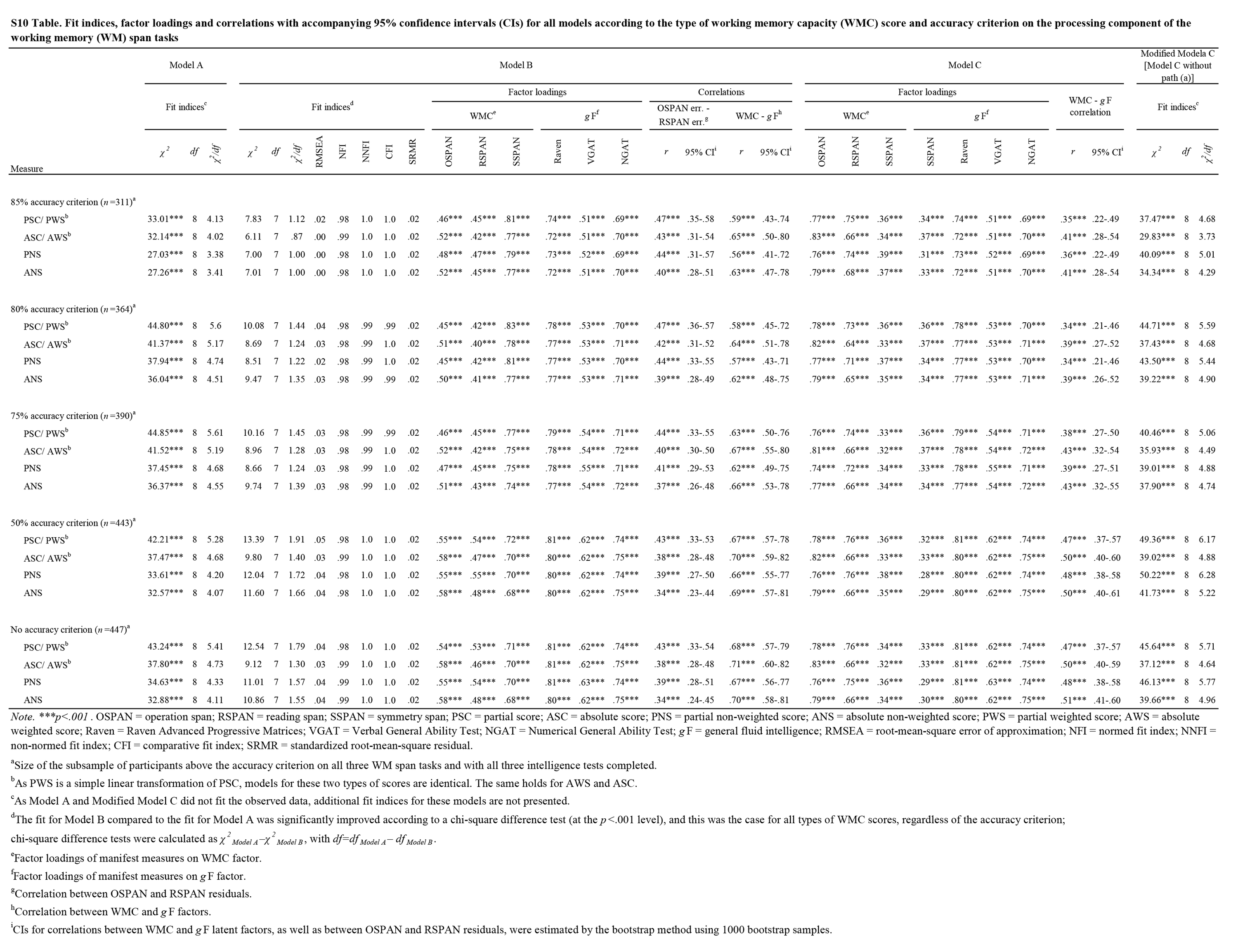

Supplement: S10 Table — (TIF) [file pone.0205169.s010.tif]

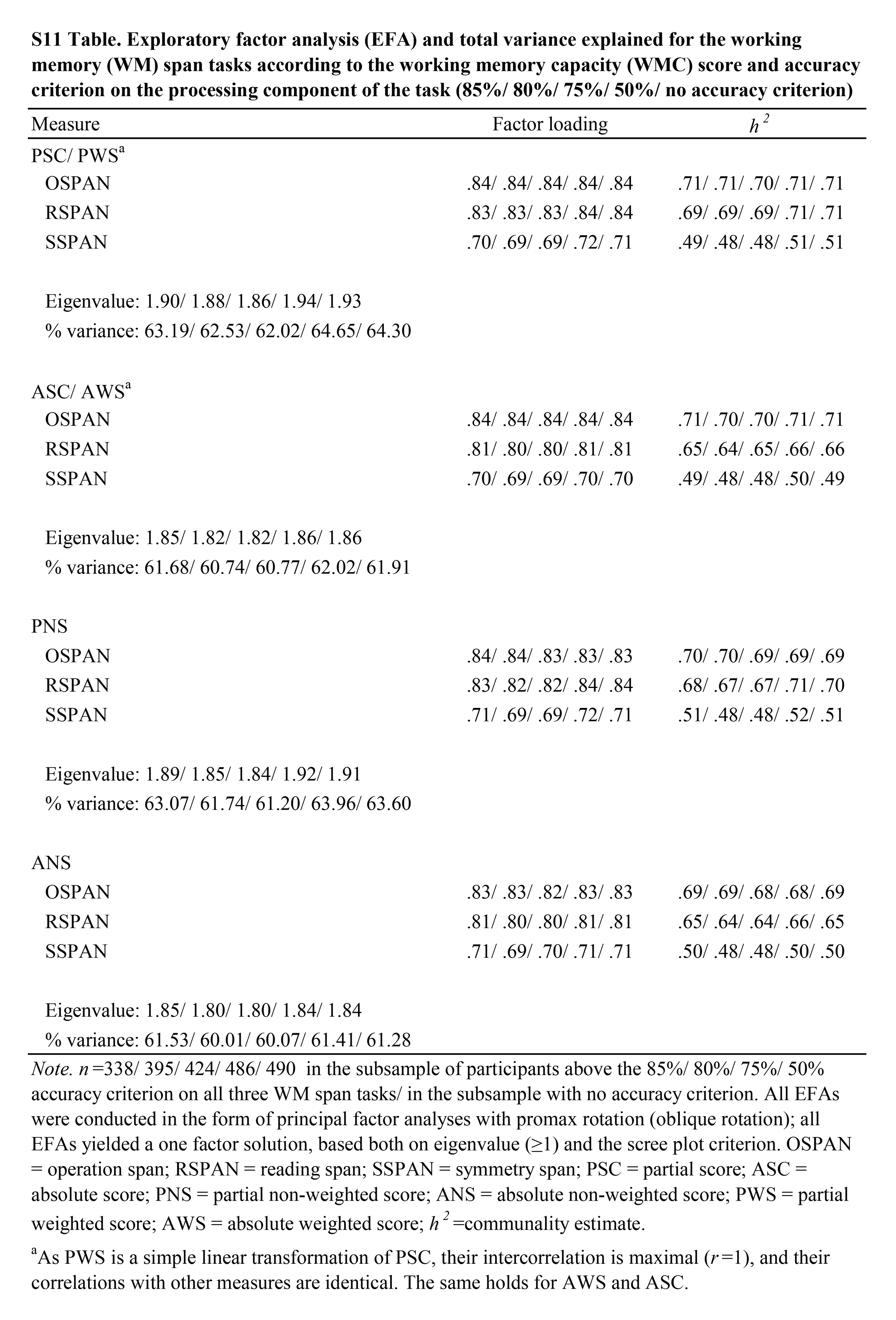

Supplement: S11 Table — (TIF) [file pone.0205169.s011.tif]

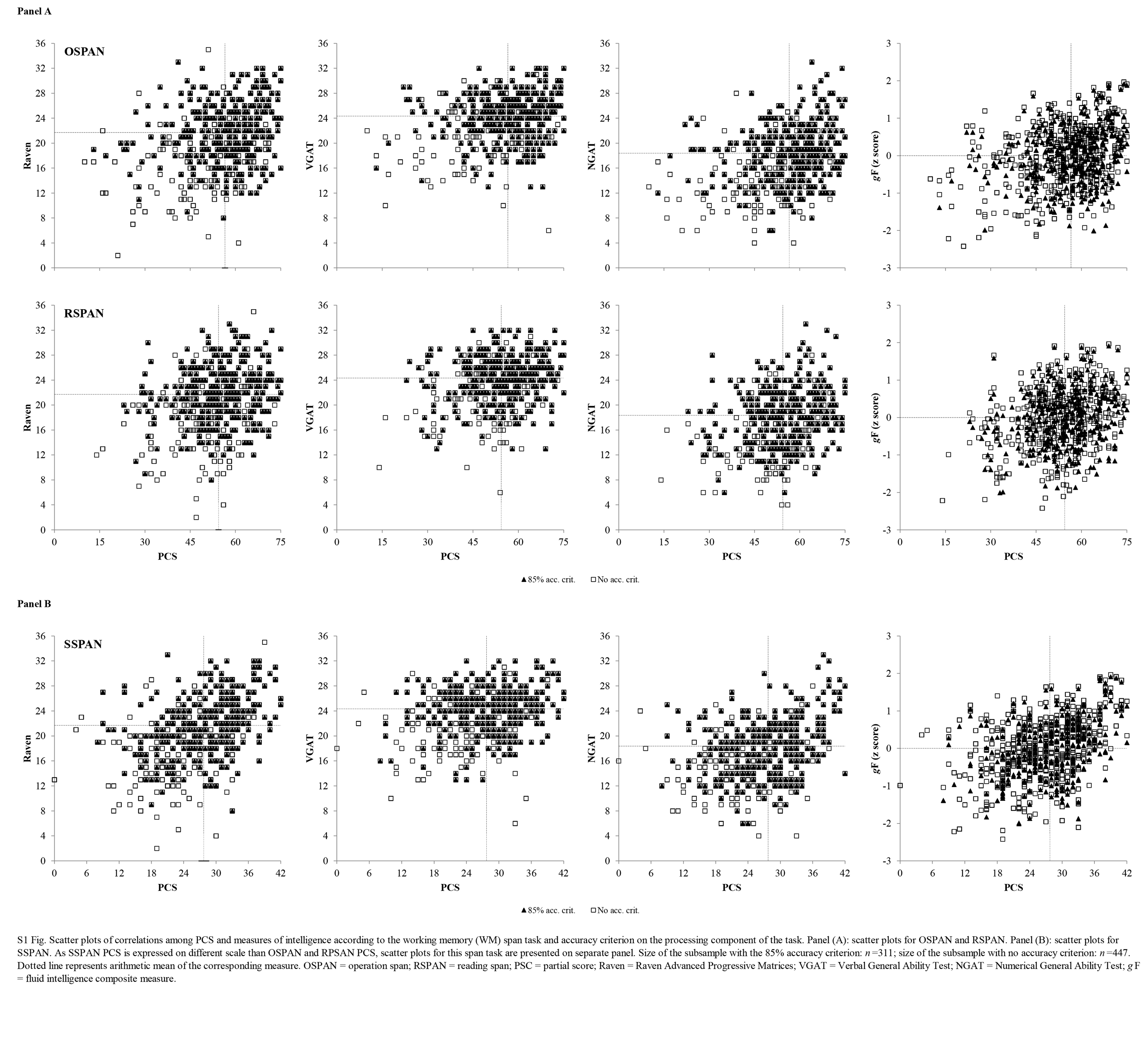

Supplement: S1 Fig — Panel (A): scatter plots for OSPAN and RSPAN. Panel (B): scatter plots for SSPAN. (TIF) [file pone.0205169.s012.tif]
